# Longitudinal dynamics of gut bacteriome and mycobiome interactions pre- and post-visceral surgery in Crohn’s disease

**DOI:** 10.3389/fcimb.2023.1275405

**Published:** 2024-01-15

**Authors:** Simon Wetzel, Alexander Müller, Eva Kohnert, Negin Mehrbarzin, Roman Huber, Georg Häcker, Clemens Kreutz, Ann-Kathrin Lederer, Mohamed Tarek Badr

**Affiliations:** ^1^Institute of Medical Microbiology and Hygiene, Medical Center–University of Freiburg, Faculty of Medicine, University of Freiburg, Freiburg, Germany; ^2^Center for Complementary Medicine, Department of Medicine II, Medical Center—University of Freiburg, Faculty of Medicine, University of Freiburg, Freiburg, Germany; ^3^Institute of Medical Biometry and Statistics, Faculty of Medicine and Medical Center, University of Freiburg, Freiburg, Germany; ^4^Centre for Biological Signaling Studies (BIOSS), University of Freiburg, Freiburg, Germany; ^5^Department of General, Visceral and Transplant Surgery, University Medical Center of the Johannes Gutenberg University, Mainz, Germany; ^6^Aufdecken gemeinsamer Prinzipien immunvermittelter Erkrankungen: von der Grundlagenwissenschaft zu neuen Therapien (IMM-PACT)-Program, Faculty of Medicine, University of Freiburg, Freiburg, Germany

**Keywords:** bacteriome, mycobiome, Crohn’s disease, visceral surgery, microbiota, cluster analysis, bacteriome-mycobiome interaction, next-generation sequencing

## Abstract

**Introduction:**

Alterations of the gut microbiome are involved in the pathogenesis of Crohn’s disease (CD). The role of fungi in this context is unclear. This study aimed to determine postoperative changes in the bacterial and fungal gut communities of CD patients undergoing intestinal resection, and to evaluate interactions between the bacteriome and mycobiome and their impact on the patients’ outcome.

**Methods:**

We report a subgroup analysis of a prospective cohort study, focusing on 10 CD patients whose fecal samples were collected for bacterial 16S rRNA and fungal ITS2 genes next-generation sequencing the day before surgery and on the 5th or 6th postoperative day.

**Results:**

No significant differences in bacterial and fungal diversity were observed between preoperative and postoperative stool samples. By in-depth analysis, significant postoperative abundance changes of bacteria and fungi and 17 interkingdom correlations were detected. Network analysis identified 13 microbial clusters in the perioperative gut communities, revealing symbiotic and competitive interactions. Relevant factors were gender, age, BMI, lifestyle habits (smoking, alcohol consumption) and surgical technique. Postoperative abundance changes and identified clusters were associated with clinical outcomes (length of hospital stay, complications) and levels of inflammatory markers.

**Conclusions:**

Our findings highlight the importance of dissecting the interactions of gut bacterial and fungal communities in CD patients and their potential influence on postoperative and disease outcomes.

## Introduction

Crohn’s disease (CD) is a severe, chronic inflammatory condition primarily affecting the terminal ilium. CD is associated with irreversible intestinal damage of the entire gastrointestinal tract, substantially impairing patient’s quality of life (Torres et al., 2017). Genetic, epigenetic, immunological, and microbiological factors are involved in the pathogenesis of CD, in conjunction with exposure to trigger factors such as smoking, diet, stress, and infections. Recent research indicates an association of the gut microbiome with CD pathogenesis, maintenance and progression. Since the early 2000s, it has been hypothesized that the overgrowth of certain gut microbes such as *Mycobacterierum avium* and adherent-invasive *Escherichia coli* (AIEC) may be involved in the pathogenesis of CD (Bull et al., 2003; Naser et al., 2004; Autschbach et al., 2005; Kotlowski et al., 2007). More recent evidence suggests that gut microbiome dysbiosis can be the cause or consequence of CD, or a combination of both, and that CD patients typically have lower complexity of their commensal gut community (Frank et al., 2007). This may enhance inflammatory and genotoxic effects of microbiota-produced metabolites (Manichanh et al., 2012; Cao et al., 2022; Zheng et al., 2022). *Faecalibacterium prausnitzii*, a member of the Firmicutes phylum, has been postulated to exhibit anti-inflammatory effects on the intestinal community in CD and to directly enhance the risk of recurrence in CD patients after bowel resection when its preoperative abundance is low (Sokol et al., 2008). *Clostridium* cluster IV is known to have anti-inflammatory effects on the intestinal microbiome and promote its homeostasis and was found to be decreased in patients with inflammatory bowel disease (IBD) (Ricanek et al., 2012; Sha et al., 2013; Li et al., 2019; Ryan et al., 2020). Although only 0.1 percent of microbial sequences detected in the human gut are of fungal origin (Qin et al., 2010; Arumugam et al., 2011), fungal communities represent a substantial part of the human gut communities as fungi are about 100 times larger than bacteria (Underhill and Braun, 2022). While fungi have been suggested to play a role in IBD pathogenesis (Standaert-Vitse et al., 2009; Chehoud et al., 2015; Liguori et al., 2016; Limon et al., 2019), their association with the various clinical features and treatment options of CD patients has not been investigated thoroughly. Previous studies have suggested a role to *Candida tropicalis* in CD dysbiosis, and its abundance has been found to be associated with anti-Saccharomyces cerevisiae antibodies (ASCA), a CD biomarker (Vermeire et al., 2001). Other groups have reported that *Malassezia* spp. have higher abundances in mucosal samples of CD patients and may be associated with the exacerbation of Crohn’s colitis (Limon et al., 2019; Ha et al., 2020). The role of fungi in CD patients who have undergone surgery is mostly unclear. A recent study suggests that in patients who underwent surgery, the cultivation rate of *Candida albicans* from intraoperative tissue samples of the peritoneal cavity is higher in patients with CD (Šašala et al., 2020).

Although surgery is not the initial choice of CD treatment, close to 50% of patients develop complications during the course of the disease that require surgical intervention within 10 years of diagnosis (Torres et al., 2020). Despite high response rates (50-70%) to current immunomodulatory drug therapies, such as monoclonal antibodies (Vasudevan et al., 2017; Guberna et al., 2021), sparing surgical resection in case of complications related to highly inflamed bowel segments remains a frequently inevitable treatment option (The German Society for Gastroenterology and Digestive and Metabolic Diseases (DGVS), 2021). In the context of surgical resection, patients commonly receive peri- and intraoperative antibiotic treatment, potentially affecting the already disturbed gut microbiome of CD patients. Furthermore, every surgical resection is a massive stress event for the human body and causes metabolic, endocrinologic and immunologic responses that may also affect the gut microbiome (Guyton and Alverdy, 2017; Lederer et al., 2021). Postoperative microbial changes may influence the development of complications and patient outcome (Qin et al., 2010; Schmitt et al., 2019; Lederer et al., 2021), but data about the postoperative gut mycobiome changes in CD patients are lacking. In this study, we first aimed to determine postoperative changes in the bacterial and fungal gut community of CD patients undergoing surgical resection therapy. Our secondary objectives were to assess patients’ outcome in relation to alterations in the gut community, OP techniques and anti-infective treatment. We further evaluated interactions between the bacteriome and mycobiome of CD patients.

## Materials and methods

### Patient cohort

This subgroup analysis investigates results from all patients suffering from CD, of a prospective cohort trial, which was performed at the Department for General and Visceral Surgery, University Medical Center Freiburg from April 2018 to August 2020. We evaluated the postoperative alteration of the gut microbiome in CD patients undergoing colorectal surgery. Inclusion criteria were age above 18 years, elective left or right hemicolectomy, sigmoid resection, deep anterior rectal resection, ileocecal resection or restoration of continuity without ostomy creation. Emergency surgeries were not considered. Before inclusion in the study, written consent was obtained from all patients after a detailed consultation. The study was approved by the local ethical committee, Medical Center—University of Freiburg, (EK-FR: 535/17) and was registered at the German Clinical Trial register (DRKS00014059).

All patients included in the study were planned to receive a standardized perioperative treatment comprising: 1) Postoperative fast-track treatment with rapid mobilization, sufficient analgesia, shortened fasting and avoidance of drains, urine catheter or nasogastric tube (Schwenk, 2022). 2) Intraoperative antibiotic prophylaxis with 2,000 mg cefazolin and 500 mg metronidazole. 3) Postoperative treatment with pantoprazole 40 mg (once a day, in the evening). 4) Analgesia with an epidural anesthesia with ropivacaine (0.2%) and sufentanil (25 µg) and metamizole (1 g, four times a day), after removal of sufentanil treatment with oxycodone (10 to 20 mg, twice a day), if necessary.

Before surgery, patients had a free choice of diet. Postoperatively, all patients started with water and tea a few hours after surgery, followed by soup, mush, porridge and yoghurt on the first postoperative day. If this was well-tolerated, patients received easily digestible food (white bread with jam, rusk, smashed potatoes, steamed vegetables, noodles, rice, low fat meat) on the second or third postoperative day. If this was also well-tolerated, patients were allowed to return to all types of foods. Pre-existing medication was continued postoperatively.

### Clinical data collection

Data were obtained prospectively by evaluation of the electronic patient’s chart and by interviews on the 3^rd^, 6^th^ and 9^th^ postoperative day. Data were recorded in paper-based case report forms and were transferred to a standard electronic table after completion of the trial. Blood samples for evaluation of inflammatory parameters were examined by the Central Laboratory of the University Medical Center of Freiburg as part of the postoperative standard procedure. Methods had been validated previously.

The clinical data contained general patient information (sex, age, BMI, performed surgery, pre-existing illnesses (cancer, diabetes, nephropathy), abuse of nicotine/alcohol/drugs, diet, previous surgery, usage of antibiotics (pre- and perioperatively), duration of hospital stay as well as frequency and kind of postoperative complications assessed by the Clavien-Dindo score (Dindo et al., 2004; Clavien et al., 2009).

### 16S rRNA and ITS2 genes amplicon library construction and sequencing

Fecal samples were collected in a special stool collector preoperatively (on the last day before surgery) as well as on the 5^th^ or 6^th^ (depending on bowel movement) postoperative day. Fecal samples were frozen immediately and stored at -80°C after receipt. Bacterial and fungal DNA extraction was performed with the ZymoBIOMICS DNA Mini Kit (Zymo Research, Irvine, CA) and additional bead beating on a FastPrep-24 homogenizer (MPBiomedicals, Santa Ana, CA). V3-4 region gene libraries were constructed with dual indexing using the *Klindworth et al.* (2013) primer pair 341F (5′- CCT ACG GGN GGC WGC AG -3′) and 805R (5′- GAC TAC HVG GGT ATC TAA TCC -3′) with 20 PCR cycles for bacterial DNA amplification and 10 PCR cycles for subsequent biochemical barcode addition (Klindworth et al., 2013). The final library was sequenced using the MiSeq v2 reagent kit (500 cycles) (Illumina Inc., San Diego, CA, USA).

ITS region 2 (ITS2) gene libraries were constructed according to the dual indexing strategy of *Kozich et al.* (2013) utilizing the gITS7 (forward: GTGARTCATCGARTCTTTG) described in *Ihrmark et al.* (2012) and ITS4ngs (reverse: TTCCTSCGCTTATTGATATGC) primers (described in *Tedersoo et al., 20*15), which anneal to the 5·8S and LSU rRNA genes flanking the ITS2 region (Ihrmark et al., 2012; Kozich et al., 2013; Tedersoo et al., 2015). PCR amplification was done with the following conditions: 30 s at 98°C; 35-40 cycles of 9 s at 98°C, 30 s at 56°C, and 30 s at 72°C; final extension for 10 min at 72°C, using the Phusion^®^ Hot Start II DNA High-Fidelity DNA Polymerase. In parallel to negative controls, a standard bacterial and fungal mock community (Zymo Research, Irvine, CA) was used as a positive control in all PCRs and sequencing runs. PCR products were enzymatically purified, and barcodes containing Illumina sequencing adapters were added in a second PCR reaction using the Quick-16S NGS Library Prep Kit (Zymo Research, Irvine, CA).

PCR products were quantified on a 1.5% agarose gel and Qubit 4.0. fluorometer (Thermo Fisher Scientific, Waltham, MA, USA), then pooled to generate equimolar subpools. Where required, the final pooled library was extracted from agarose gels with the Qiagen MinElute Gel Extraction Kit (Qiagen GmbH, Hilden, Germany), then purified with Select-a-Size DNA Clean & Concentrator (Zymo Research, Irvine, CA) according to the manufacturer’s protocol. Pooled libraries were quantified by a NEBNext library quantification kit (New England BioLabs GmbH, Frankfurt am Main, Germany) and analyzed on a QiaXcel advanced system (Qiagen, Hilden, Germany). The final library was sequenced using the MiSeq v2 reagent kit (500 cycles) (Illumina Inc., San Diego, CA, USA) on a MiSeq system with 10-20% PhiX spike-in.

### Sequencing data and statistical analysis

Raw fastq files read quality was assessed using FastQC. Further quality control, trimming, and analysis of short-reads were done using the DADA2 analysis pipeline and visualized using multiple packages in the R programming language in a Linux environment. Amplicon sequence variants (ASVs) were extracted from DADA2 and were assigned to taxonomy ranks using the UNITE and GTDB databases (Kõljalg et al., 2020; Rinke et al., 2021). Testing for bacterial beta diversity was performed by non-metric Multidimensional Scaling (NMDS), measuring Bray-Curtis Dissimilarity Distances. Perioperative differential abundances assessed by alpha and beta diversity were tested for statistical significance by Wilcox and Adonis testing. LEfSe analysis (linear discriminant analysis effect size) was performed to identify bacterial and fungal genera as discriminative features to distinguish between classes (pre-/post-surgical samples). Mycobiome-bacteriome interactions were conducted by comparing relative bacterial and fungal abundances by the Spearman method using the *corrplot* package in R programming language, and the interactions were additionally assessed by network and cluster analysis using *SpiecEasi* R package and Louvian clustering. The sequencing data obtained were correlated with different parameters of the clinical data using correlation and plotting packages in the R programming language. For statistical testing, no FDR correction was performed.

## Results

### Characteristics of the study cohort

Overall, 10 patients who underwent abdominal surgery due to CD were evaluated. Sex of the included patients was well-balanced (50% male, 50% female). Patients were on average 38.9 ± 15.9 years old (range 18-62 years). The average BMI of patients was 25.0 ± 5.7 kg/m^2^ (range 19-32 kg/m^2^). Nicotine, alcohol and drug addiction was negated by all patients. Six patients stated to consume alcohol occasionally. All patients were on an omnivorous diet. More than half of the patients (n = 6, 60%) had abdominal surgery for the first time. Surgery was performed laparoscopically in 20% of patients (n = 2). Besides CD, just one patient (10%) suffered from another pre-existing illness (hypertension). The indication for surgery was mostly stenosis (n = 6, 60%). Patients had been suffering from CD for on average 13.7 ± 12.7 years (range 1-34 years). Previous medications included prednisone, azathioprine and biologics (ustekinumab, infliximab, adalimumab). A detailed overview of each patient is shown in **Table 1**. Overall, four patients developed postoperative complications. Three patients had a mild postoperative complication (classified as Clavien-Dindo I) due to paralytic ileus. One patient received a CT scan due to poor clinical condition and severe paralytic ileus showing abdominal fluid collections with necessity of drainage (classified as Clavien-Dindo III). None of the patients died. Duration of hospital stay was on average 8.5 ± 2.1 days (range 6-13 days).

**Table 1:** Detailed overview of every included patient – demographic and surgery-related data.

Pa.-No.	Sex	Age (years)	BMI (kg/m^2^)	Year of Diagnosis	Disease activity	Medication*	Smoking/Alcohol	Diet	Origin
1	m	51	20.8	2008	none	prednisone	no/no	omni	Germany
2	m	32	31.5	2006	abdominal discomfort	prednisone	no/no	omni	Germany
3	f	62	20.6	1986	abdominal pain, diarrhea	adalimumab	yes/occ.	omni	Germany
4	f	30	19.1	2010	abdominal pain	prednisone, adalimumab	no/occ.	omni	Germany
5	f	18	20.5	2015	abdominal pain, constipation	none	no/occ.	omni	Germany
6	m	59	27.2	1985	abdominal pain, diarrhea	none	yes/no	omni	Italy
7	f	51	28.3	n/a	none	budesonide	no/no	omni	n/a
8	m	32	25.6	1994	abdominal discomfort	infliximab	no/occ.	omni	Germany
9	m	18	20.5	2018	none	none	no/occ.	omni	Germany
10	f	36	36.0	2016	extra-abdominal	infliximab	yes/occ.	omni	Germany
Pa.-No.	Surgery		Approach	Reason for surgery	Previous abdominal surgery	CRP Pre/Post-OP (-1/+3/+6 days) [mg/dL]	Leucocytes Pre-/Post-OP (-1/+3/+6 days) [Thousands/µl]		Compli-cations**
1	continuity restoration		open	restoration of continuity	ileocoecal resection	3.1/xx/84.9	6.10/7.04/7.41		I (paralytic ileus)
2	ileocoecal resection		open	stenosis	none	13.5/xx/19.0	17.55/14.84/13.37		I (paralytic ileus)
3	right hemi-colectomy		open	stenosis	ileocoecal resection	2.9/xx/21.3	4.29/4.53/5.03		none
4	continuity restoration		open	restoration of continuity	ileocoecal resection	2.9/xx/39.4	14.16/9.50/9.09		none
5	ileocoecal resection		lap	stenosis	none	28.3/xx/14.4	5.81/5.14/5.19		none
6	right hemi-colectomy		open	stenosis	right hemi-colectomy	2.9/xx/50.6	7.67/7.54/7.14		none
7	left hemi-colectomy		open	stenosis	none	14.7/xx/160.4	5.10/12.36/8.75		IIIa (^+^)
8	procto-colectomy		open	dysplasia	none	3.5/xx/18.1	6.71/8.03/6.80		none
9	continuity restoration		open	restoration of continuity	right hemi-colectomy	3.9/xx/19.5	7.06/7.97/4.78		I (paralytic ileus)
10	lleocoecal resection		lap	stenosis	none	x/x/36.9	5.88/7.15/4.48		none

(*recent medication due to Crohn’s disease, n/a = not available, occ. = occasionally; omni = omnivorous diet, lap = laparoscopic, **according to the Clavien-Dindo classification, ^+^placement of drainage due to abdominal fluid collections).

### Generation of 16S rDNA and fungal ITS2 amplicon sequencing data

Seventeen fecal samples were evaluated (8 preoperative samples and 9 postoperative samples). For eight patients, both a preoperative and a postoperative sample was available for evaluation. In two patients no fecal sample could be obtained within 24 hours before surgery (patients 4 and 8), and one patient was discharged from the hospital before collection of a postoperative sample was possible (patient 8). Patients without collected pre- and postoperative samples were excluded from comparative analyses between pre- and postoperative state (LEfSe analysis, grouped analysis of microbial abundance changes and subsequent clinical data correlation) to avoid methodological bias. From all samples, bacterial DNA was successfully extracted. After amplification of the V3-4 region, all samples were found suitable for NGS sequencing.

After preliminary evaluation and comparison with different primer sets, the primers of *Ihrmark et al.* were selected for fungal ITS sequencing and tested for their sensitivity in amplifying fungal DNA. The successful amplification of fungal DNA in 16 of 17 samples (94%) suggest that the primers used by *Ihrmark et al.* are appropriate for the identification of fungi in fecal samples of CD patients.

Bacterial 16S sequencing of 17 samples generated 519,656 reads, with an average of 30,568 reads per sample. After fungal ITS2 sequencing of 16 samples 612,948 raw reads were generated, averaging 38,309 reads per sample. The overall quality of fungal sequencing data is illustrated in **Supplementary Figure 2** with a mean Phred score (Q-score) of greater than 30.

### Gut bacterial and fungal composition in Crohn’s disease differ pre- and post-surgery

**Figure 1:**
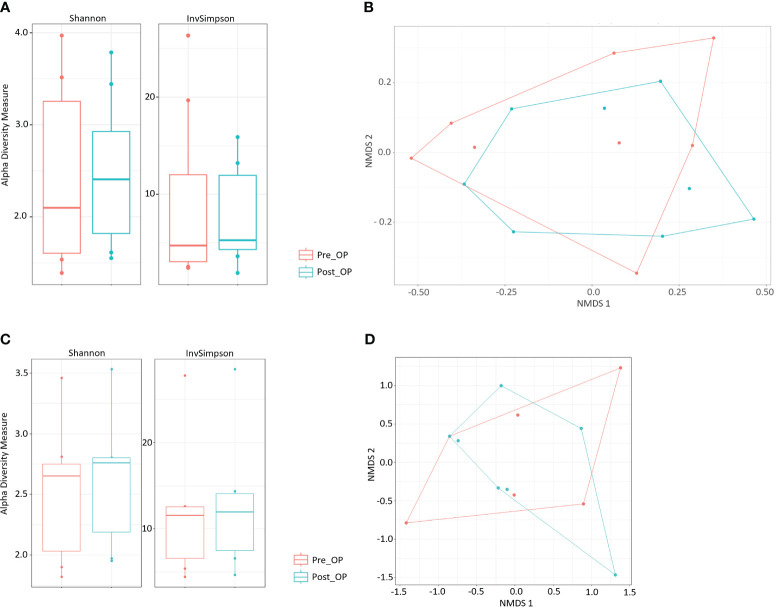
Pre- to postoperative changes of bacterial gut communities. **(A)** Bacterial alpha diversity of pre- and postoperative fecal samples. **(B)** NMDS based on Bray-Curtis distances showing different bacterial community composition across pre- and postoperative fecal samples. **(C)** Fungal alpha diversity of pre- and postoperative fecal samples. **(D)** NMDS based on Bray-Curtis distances showing different fungal community composition across pre- and postoperative fecal samples.

Analysis of bacterial 16S rDNA data revealed no significant alterations in bacterial alpha diversity in postoperative samples (p=0.89) (**Figure 1A**). Testing for bacterial beta diversity with NMDS showed clustering of pre-and postoperative community compositions between CD patients (**Figure 1B**, p=0.17). Bacterial principal coordinate analysis (PCoA) and bacterial NMDS analysis with labelling of identified bacterial phyla and surgical status are presented in **Supplementary Figures 3A** and **8**. In more detail, pre- and postoperative samples were analyzed by their bacterial relative abundance on different taxonomic levels. Postoperative changes in the relative abundance of the top 100 most abundant bacterial amplicon sequence variants (ASVs) were identified (**Supplementary Figures 1A, B**). A postoperative increase was detected for the genera *Akkermansia, Bifidobacterium, Clostridium, Coprococcus, Gemmiger, Enterococcus, Enterobacter, Eubacterium, Morganella, Parabacterium, Roseburia* and *Ruminococcus*. A decrease in postoperative samples was found for bacteria belonging to the following genera: *Bacteroides, Blautia, Dorea, Escherichia, Faeclibacterium, Klebsiella, Lactococcus, Megasphaera, Streptococcus, Veilonella* and *Lactobacillus*. Violin plots of pre- and postsurgical bacterial relative abundances on class, genus and species level are illustrated in **Supplementary Figures 4**-**6**.

The fungal alpha diversity did not change significantly in postsurgical samples of CD patients (p=0.62), but a trend towards increased fungal alpha diversity was detected (**Figure 1C**). NMDS analysis revealed no significant differences in fungal beta diversity of pre- and postoperative group clusters (p=0.942, **Figure 1D**). Mycobial PCoA and NMDS analysis with labelling of identified mycobial genera and surgical status are shown in **Supplementary Figures 3B** and **9**. *Candida* and *Saccharomyces* were among the most abundant genera in all fecal samples. On genus level changes in the relative abundance of different fungi genus were detected, particularly a postoperative increase of *Candida* (**Supplementary Figure 7**) and *Clavispora* and decrease of *Saccharomyces* and *Penicillium* (**Supplementary Figures 1C, D**).

### Bacterial and fungal biomarkers distinguish Crohn’s patients’ surgical status and interact with their clinical phenome

**Figure 2:**
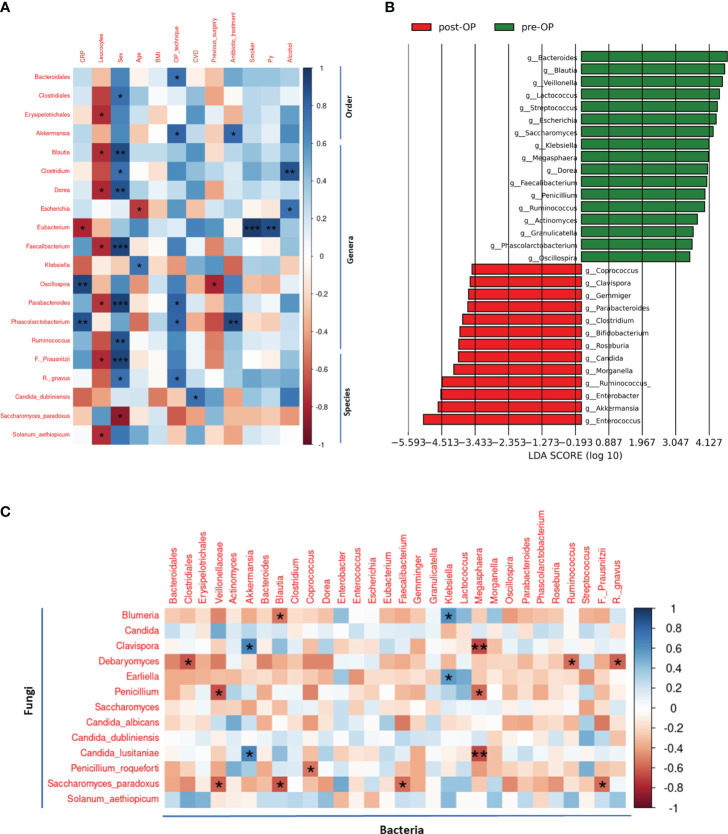
Pre- and postoperative classification feature analysis using LEfSe and correlation analysis of clinical data with preoperative bacterial and fungal communities and interkingdom correlations **(A)** Preoperative bacterial and fungal communities correlated with preoperative clinical features. Only bacterial and fungal taxonomic ranks (order, genus, species) with at least one significant correlation (p<0.05) are shown. Correlation analysis was done using the Spearman method and clinical data was correlated with relative abundances on different taxonomic levels. **(B)** Linear discriminant analysis Effect Size (LEfSe) analysis on bacterial and fungal genus level with operative status (pre-/postoperative) as class. Genera identified as features of preoperative samples are highlighted in green and of postoperative samples in red. Visualized are the top 30 features identified with an LDA effect size >3. Lefse analysis was performed using LEfSe analysis pipeline and package by Huttenhower Lab. **(C)** Correlation analysis of most abundant fungal and bacterial communities in pre- and postoperative samples. Spearman method was used for correlation measurement. *: p<0.05, **: p<0.01, ***: p<0.001.

Ordinal Spearman statistical analysis revealed correlations between the pre-operative bacterial and fungal gut composition of patients with Crohn’s Disease (CD) and clinical data collected before surgery (**Figure 2A**). Levels of inflammatory markers (CRP and leukocytes) were associated with ten fungal and bacterial taxonomic ranks in the samples of preoperative stool. Low preoperative abundances of *Erysipelotrichiales, Blautia, Dorea, Faecalibacterium, Parabacteroides* and *Solanum aethoipicum* correlated positively with a higher preoperative leukocytes count. Higher pre-surgical CRP levels were found in CD patients with higher relative abundances of the *Oscillospira* and *Phascolarctobacterium*, whereas lower pre-surgical CRP levels were associated with higher relative abundances of the *Eubacterium* genus. Previous abdominal surgery was associated with detected lower abundances of *Oscillospira* in presurgical samples. Female patients in this CD cohort had higher preoperative relative abundances of multiple bacterial taxonomic ranks, indicating different predominant bacterial organisms in the dysbiotic gut community of female compared to male patients. In younger CD patients, higher bacterial abundances of *Escherichia* (r=-0.750, p= <0.001) and lower abundances of *Klebsiella* (r=0.524, p=0.037) were detected. Cigarette smoking was strongly associated with a higher relative abundance of the *Eubacterium* genus (r=0.660, p=0.005). Higher relative abundances of the *Clostridium* and *Escherichia* genera were detected in CD patients who occasionally consumed alcohol.

Linear discriminant analysis Effect Size (LEfSe) analysis identified the top 30 bacterial and fungal genera as discriminative features to distinguish between classes (pre-/post-OP samples) with an LDA effect size >3 (**Figure 2B**). As most discriminative features of the preoperative class *Bacteroides, Blautia, Veillonella, Lactococcus, Streptococcus, Escherichia and Saccharomyces* were identified. For the postoperative class *Enterococcus, Akkermansia, Enterobacter, Ruminococcus, Morganella* and *Candida* were detected as most discriminative features.

### Crohn’s disease is associated with interkingdom correlations

Results of the Spearman correlation analysis of fungal and bacterial taxa (**Figure 2C**) varying most in their pre- to postoperative abundance revealed 13 significant negative correlations, implying strong associations in growth and abundance of these fungi and bacteria in CD patients.

Four positive interkingdom correlations were identified as possible commensal fungi and bacteria in CD patients. On a significant level, the bacterial genus *Klebsiella* correlated positively with the two fungal genera *Blumeria* and *Eariella* and the bacterial genus *Blautia* correlated positively with the fungal genus *Clavispora* and fungal species *Candida lusitaniae*.

**Figure 3:**
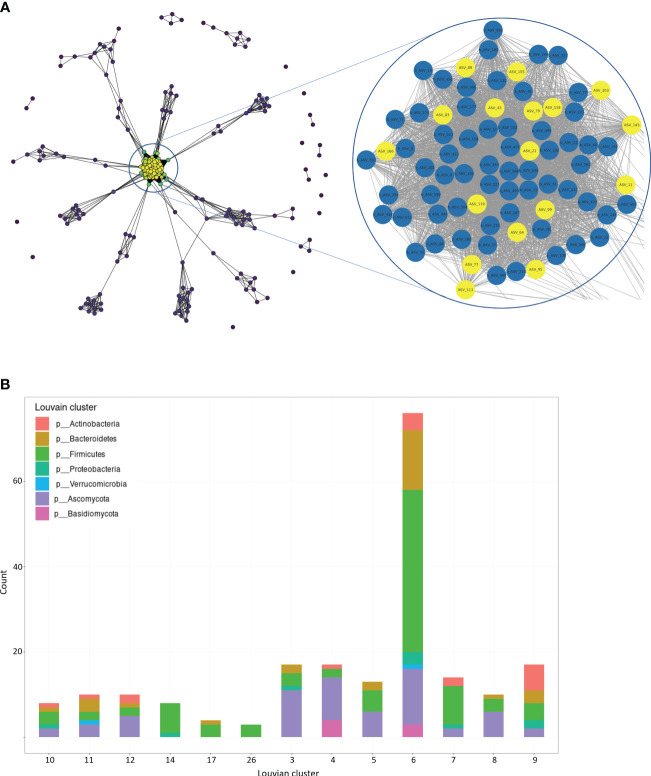
Network and Cluster Analysis of bacteriome and mycobiome communities in CD patients. **(A)** Bacterial and fungal network: The degree distribution of the complete network (left) is visualized by the node color, representing the degree value of each node (blue [low] to yellow [high]). The network center shows a region with nodes of very high degree value. The representation of the network center reveals strong correlations between multiple bacterial ASVs (blue) and fungal ASVs (yellow) with a degree value >48 of each node (right). Edges with very low weight (<0.01), negative edges and unconnected vertices were removed prior to visualization. **(B)** Louvian Clustering Network: Louvian Clustering was performed for bacterial and fungal community detection and 13 main clusters were identified. 10 of 13 identified clusters contain bacterial and fungal species, represented by bacterial and fungal phyla in bar plot. Cluster 6 represents the largest cluster of the network (yellow cluster in **Figure 4A**). Further network plots are provided in **Supplementary Figures 9**-**11**. Detailed clustering results are listed in **Supplementary Table 1**.

Network analysis of all identified fungal and bacterial ASVs revealed multiple further interactions in the gut community of CD patients (**Figure 3A**, left panel). In the network center, multiple fungal and bacterial ASVs were detected with particularly strong interactions (degree value of >48 of each ASV) (**Figure 3A**, right panel). By using the Louvian clustering method in total 13 clusters were identified (**Supplementary Table 1**). Ten of 13 clusters revealed interactions of fungal and bacterial ASVs with cluster 6 (network center) as the largest cluster among them (**Figure 3B**). Of all 6396 identified ASVs (6221 bacterial and 175 fungal), network and cluster analysis identified 207 (3.2%) fungal and bacterial ASVs grouped into 13 clusters that exhibited strong interactions. Plots from individual mycobial and bacterial interaction network analysis are presented in **Supplementary Figures 10** and **11**. Mycobiome-bacteriome interaction network analysis with labeling of bacterial and mycobial organisms on genus level is shown in **Supplementary Figure 11**.

### Mycobiome-bacteriome interactions shape post-surgery disease outcome in Crohn’s patients

**Figure 4:**
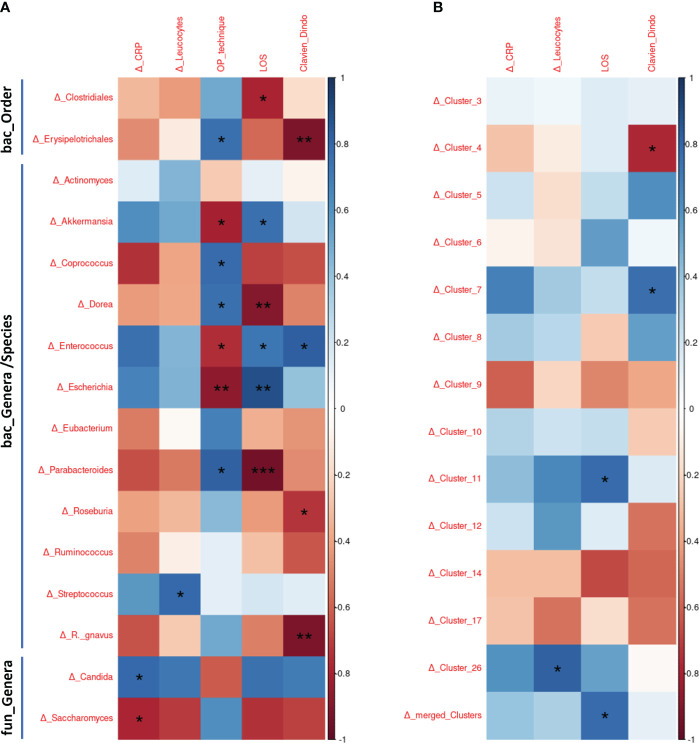
Correlation of surgery-associated alterations in the gut bacteriome and mycobiome with patient outcome, inflammatory parameters and surgical technique. **(A)** Correlation analysis of pre-to-postsurgical relative bacterial and fungal abundance changes with clinical and patient outcome data. Delta (Δ) sign represents relative abundance changes of the respective taxonomic rank between pre-and postoperative samples of each patient and represents changes of pre- and postoperative inflammatory markers. **(B)** Correlation analysis of surgery-associated relative abundance changes of the 13 identified fungal and bacterial clusters with clinical and patient outcome data. All 13 clusters were additionally merged for analysis. Delta (Δ) sign represents relative abundance changes of the respective clusters between pre-and postoperative samples by each patient and represents changes of pre- and postoperative inflammatory markers. The scale bar represents the Spearman correlation coefficient, ranging from -1 (red, maximal inverse correlation) to +1 (blue, maximal positive correlation) as a measurement of the relationship between two variables. *: p<0.05, **: p<0.01, ***: p<0.001.

Analysis of post-surgical alterations to the bacteriome and mycobiome in CD patients demonstrated a connection between changes in gut communities and disease outcomes, complication rates, and inflammation markers (**Figure 4A**). A decrease of *Saccharomyces* and increase of *Candida* in the post-surgical mycobiome of CD patients was found to correlate with higher postoperative CRP levels. Increased relative amounts of *Streptococcus* were associated with increased post-surgical leucocytes levels. Correlation analyses between surgical technique used and changes in the postoperative gut community revealed that open surgery was associated with increased relative abundances of *Akkermansia, Enterococcus*, and *Escherichia*, while laparoscopic surgery was associated with increased postoperative abundances *of Erysipelotrichiales, Coprococcus, Dorea*, and *Parabacteroides* in CD patients.

The length of hospitalization (LOS) was found to be shorter in patients with postoperative changes to higher relative abundances of *Clostridiales, Dorea* and *Parabacteroides*. Increased relative abundances of *Akkermansia, Enterococcus* and *Escherichia* after surgery was associated with a longer LOS. Patients with increased postoperative relative abundances of *Erysipeltrichales, Roseburia*, and *Ruminococcus gn*avus experienced less severe post-surgical complications as measured by the clinical Clavien-Dindo score. The reported associations of LOS and post-surgical complications with postoperative abundance alterations in the microbial profile on genus level should be considered as preliminary findings due to the small number of individual patients.

Examination of postoperative abundance changes of the identified bacterial and fungal clusters showed that the interactions between the two kingdoms are associated with disease outcomes of CD patients (**Figure 4B**). Higher postoperative abundances of clusters 4, 7, 11 and 26 were significantly associated with longer LOS, higher complication rates (despite cluster 4), and increased post-surgical inflammatory markers (leucocytes). By merged correlation analysis of all clusters (207 of all 6,396 ASVs [3.2%] with strong bacterial and fungal interactions) a positive correlation between increased postoperative abundances of the 207 clustered AVSs and longer LOS was detected.

## Discussion

In recent years, it has become increasingly clear that the gut microbiome plays a critical role in the development and progression of CD. Surgery, such as resection of the affected part of the intestine, is used as a treatment for stenotic or intractable CD, which might also have an impact on the gut microbiome. The present study aimed to investigate changes in the gut bacteriome and mycobiome of CD patients before and after bowel surgery and to evaluate the correlation between those changes and clinical parameters and outcomes. To our knowledge, this is the first study to investigate peri- and post-operative changes in the gut bacteriome and mycobiome in CD patients undergoing bowel surgery. Analysis of peri- and postsurgical fecal samples revealed various disparities between the bacterial and fungal communities, correlating with inflammatory markers such as CRP and leucocytes, and various comorbidities and risk factors such as smoking and alcohol consumption, suggesting their impact on disease activity. Our findings in pre-surgical samples confirm dysbiosis in CD patients, with low fungal and bacterial alpha diversity in their gut communities (Lee and Chang, 2021). In the post-surgical bacteriome, increased *Streptococcus* abundances showed a correlation with a higher postoperative leucocytes count (p<0.05). The presurgical relative abundance of the *Eubacterium* genus was found to be increased (p<0.001) in smoking CD patients and showed negative correlation with presurgical CRP levels (p<0.05). *Eubacterium* spp. were previously reported to exert anti-inflammatory effects on the gut microbiome of humans promoting short-chain fatty acid production (Barcenilla et al., 2000; Pryde et al., 2002; Macfarlane and Macfarlane, 2011). The association between higher *Eubacterium* abundances and smoking detected in this study has been previously reported in the gut microbiome of mice after smoke exposure and in the salivary microbiome of smoking humans (Duan et al., 2017; Meng et al., 2022). However, the literature is inconsistent as other studies found unchanged or decreased *Eubacterium* spp. abundances in the gut microbiome of smokers (Benjamin et al., 2012; Yan et al., 2021), indicating distinct species-level exerting effects of the *Eubacterium* genus in the context of smoking and anti-inflammation.

The detection of a higher abundances of *Candida albicans* in post-surgical samples and the positive correlation of *Candida* genus abundance changes with increased post-surgical CRP levels supports the hypothesis of a post-surgically disturbed mycobiome due to surgery. *Candida albicans* is a regular member of the healthy gut, but also one of the most common human fungal pathogens. *Saccharomyces* spp., such as *S. boulardii* and *S. cerevisiae* have been found to have beneficial effects on acute and chronic gastrointestinal diseases (Kelesidis and Pothoulakis, 2012; Sokol et al., 2017). However, *S. cerevisiae* has also been reported to be involved in decreased epithelial barrier function and increased colitis in mouse models (Chiaro et al., 2017). The lower postoperative abundance of *Saccharomyces* might indicate a therapeutic effect after bowel resection of highly inflamed bowel areas but may also be indicative of a disturbed intestinal homeostasis, as reflected by a lower abundance of beneficial *Saccharomyces* and a higher abundance of potentially pathogenic *Candida* after surgery. *Candida* and *Saccharomyces* additionally were identified as the most discriminative fungal features between pre- and post-surgical fecal samples by LEfSe analysis. These findings may suggest that visceral surgery leads to unfavorable fungal changes in the gut community of CD patients.

The impact of surgical technique on bacteriome-mycobiome interactions in CD patients is not well understood. In our patients, we see that open conventional and laparoscopic surgery correlated with different relative abundance changes of several gut members after surgery. Open conventional surgery was associated with a post-surgical increase of *Enterococcus, Escherichia* and *Akkermansia*, which are commonly associated with postoperative infections (Buffie and Pamer, 2013). Previous abdominal surgery was not associated with significant differences in the presurgical microbial profile, apart from detected lower abundances of the bacterial genus *Oscillospira*. Low levels of *Oscillospira* were found to be associated with obesity in previous 16S rRNA microbiome studies (Goodrich et al., 2014; Chen et al., 2021). In this cohort, patients who underwent repeated surgery had a higher BMI compared to patients without any history of abdominal surgery (mean BMI 28.38 kg/m^2^ [previous surgery] compared to 21.64 kg/m^2^ [no surgery]). Thus, the observed correlation between lower *Oscillospira* abundances and previous surgery might be partially explained by BMI as a confounder, although overall no significant correlation between BMI and *Oscillospira* abundances was observed in presurgical samples. In this subgroup analysis 50% of the patients were female, so both genders were equally represented. The gender correlated with differences in relative presurgical bacterial and fungal taxa abundances. A larger cohort size would allow re-evaluation of described presurgical findings of different predominant bacterial organisms in the dysbiotic gut community of female compared to male patients. Postoperative abundance increases of *Enterococcus*, *Escherichia* and *Akkermansia* were associated with longer length of hospitalization. The delayed discharge might be a consequence of more complex surgical procedures, but it is also possible that open surgical procedures in Crohn’s disease patients permit increased pathogenic fungal and bacterial growth in the gut, contributing to a delayed recovery. Open surgical procedures are typically accompanied with more complex pre-operative disease, previous operations, longer operative time, and increased intraoperative antibiotic treatment compared to minimally invasive surgery. The detected associations of postoperative microbial changes with unfavorable clinical outcomes in patients undergoing open conventional surgery support the rationale of using laparoscopic techniques in CD patients in clinical situations in which open surgery can be avoided as a last resort.

A total of 17 significant interkingdom correlations between bacterial and fungal organisms were identified in perioperative samples using the spearman correlation method. In the majority of cases - 13 out of 17 - a negative correlation between the abundance of bacteria and fungi was observed, highlighting the spatial and nutrition competition dynamics between these two kingdoms and the potential role of these competition dynamics in CD patients. Four out of 17 cases showed a positive correlation in the relative abundance of the bacterial and fungal gut members, suggesting a symbiotic relationship between these organisms. Network and cluster analysis of interkingdom interactions in the gut bacteriome and mycobiome of CD patients identified 13 bacterial and fungal interaction clusters, containing 207 ASVs (3.2% of all 6396 ASVs). Pre-to-post-surgical abundance changes (*Δ)* of 5 of 13 identified clusters were found to correlate significantly with clinical outcome parameters (LOS, Clavien-Dindo complication score) and post-surgical inflammatory marker changes of CD patients. Moreover, merging all 13 clusters revealed a significant correlation between postoperative increases in relative abundance of the 207 identified ASVs with a longer hospital stay, highlighting the importance of dissecting the interactions of gut bacterial and fungal communities and focusing on symbiotic groups of the microbiome as biomarkers for disease activity rather than singular entities of these communities, as changes in the microbiome presumably never happen independently of other community members. A continuous challenge in determining the relevance of specific microbial changes and identifying biomarkers is to assess the interaction bacterial or fungal organisms with other gut members in the context of the overall microbiome profile, thus enhancing either symbiotic or pathobiontic effects.

Our study demonstrates the value of considering the fungal community when studying the impact of various disease states and surgical interventions on the gut microbiome, and how fungal community members may be able to provide some of the missing links between the gut microbiome dynamics and diseases. Nevertheless, our study has some limitations. The relatively small sample size of our study - 10 CD patients - limits the generalizability of our findings and the robust conclusions about the relationship between the gut microbiome and mycobiome and surgical outcomes in CD patients. Another limitation of our study is that we only collected samples at two time points, before and after surgery. This does not allow us to track the changes in the gut microbiome and mycobiome over a longer period. First faecal sample collection was performed on 5^th^ or 6t^h^ postoperative day due to postoperative cost build-up with first reliable defecation after a few days. Later collection of faecal samples and the possibility of temporal resolution of postoperative microbiome changes beyond the 5^th^ and 6^th^ postoperative day was limited by early discharge, on average 8.5 days after surgery. A sample collection at more time points would have enabled a broader contextualisation of the postoperative changes observed. Due to the high turnover rate of the intestinal epithelium and its effect on receptor expression, gut integrity and immune function, it cannot be excluded that certain observed microbial changes observed on the 5^th^/6^th^ postoperative represent short-term responses of the gut microbiome due to surgical stress. Additional sampling at a later time point would allow more precise conclusions to be drawn about the observed dynamics in the postoperative gut microbiome of CD patients and facilitate the differentiation of changes in response to surgical stress from anti-inflammatory and disease-limiting effects on the microbiome by the resection of highly inflamed bowel areas. The studied cohort lacked a control group, and it is difficult to determine whether the changes in gut microbiome and mycobiome observed are specific to CD patients undergoing surgery or are a general response to surgery. However, in this CD subgroup analysis, an overall low microbial diversity was detected, supporting the reported dysbiosis theorem in CD patients with low microbial diversity compared to healthy controls (Manichanh et al., 2006; Galazzo et al., 2019), particularly in a high-activity disease state potentially leading to surgery. Further studies with larger number of study subjects and healthy controls with a longer sampling period will be needed to draw firmer conclusions.

In this study, we aimed to shed light on the interactions between the gut microbiome and surgery in the context of CD and contribute to the knowledge base for the development of better treatment strategies for CD patients. Further evaluation of the identified candidate protective fungal and bacterial genera correlating with a better outcome after surgery in CD patients together with possible fecal transplantation studies in clinical studies will be of future interest. Such studies could further uncover hidden correlations between the different microbial communities within the gut microbiome, as well as provide insights and potential new therapeutic approaches to CD disease.

## Data availability statement

The datasets presented in this study can be found in online repositories. The names of the repository/repositories and accession number(s) can be found below: https://www.ebi.ac.uk/ena, PRJEB65608.

## Ethics statement

The studies involving humans were approved by Local ethical committee of Medical Center—University of Freiburg (EK-FR: 535/17). The studies were conducted in accordance with the local legislation and institutional requirements. The participants provided their written informed consent to participate in this study.

## Author contributions

SW: Conceptualization, Data curation, Formal Analysis, Investigation, Methodology, Project administration, Validation, Visualization, Writing – original draft, Writing – review & editing, Software. AM: Conceptualization, Data curation, Methodology, Project administration, Writing – review & editing, Investigation. EK: Writing – review & editing, Data curation. NM: Methodology, Writing – review & editing. RH: Writing – review & editing, Project administration, Supervision. GH: Writing – review & editing, Project administration, Supervision. CK: Writing – review & editing, Data curation. AL: Conceptualization, Investigation, Methodology, Project administration, Resources, Supervision, Validation, Writing – review & editing, Data curation. MB: Conceptualization, Investigation, Methodology, Project administration, Resources, Supervision, Validation, Writing – review & editing, Data curation, Formal Analysis, Software.

## References

[B1] Arumugam M. Raes J. Pelletier E. Le Paslier D. Yamada T. Mende D. R.. (2011). Enterotypes of the human gut microbiome. Nature 473 (7346), 174–180. doi: 10.1038/nature09944 21508958 PMC3728647

[B2] Autschbach F. Eisold S. Hinz U. Zinser S. Linnebacher M. Giese T.. (2005). High prevalence of Mycobacterium avium subspecies paratuberculosis IS900 DNA in gut tissues from individuals with Crohn's disease. Gut 54 (7), 944–949. doi: 10.1136/gut.2004.045526 15951539 PMC1774626

[B3] Barcenilla A. Pryde S. E. Martin J. C. Duncan S. H. Stewart C. S. Henderson C.. (2000). Phylogenetic relationships of butyrate-producing bacteria from the human gut. Appl. Environ. Microbiol. 66 (4), 1654–1661. doi: 10.1128/AEM.66.4.1654-1661.2000 10742256 PMC92037

[B4] Benjamin J. L. Hedin C. R. H. Koutsoumpas A. Ng S. C. McCarthy N. E. Prescott N. J.. (2012). Smokers with active Crohn's disease have a clinically relevant dysbiosis of the gastrointestinal microbiota. Inflammation Bowel Dis. 18 (6), 1092–1100. doi: 10.1002/ibd.21864 22102318

[B5] Buffie C. G. Pamer E. G. (2013). Microbiota-mediated colonization resistance against intestinal pathogens. Nat. Rev. Immunol. 13 (11), 790–801. doi: 10.1038/nri3535 24096337 PMC4194195

[B6] Bull T. J. McMinn E. J. Sidi-Boumedine K. Skull A. Durkin D. Neild P.. (2003). Detection and verification of Mycobacterium avium subsp. paratuberculosis in fresh ileocolonic mucosal biopsy specimens from individuals with and without Crohn's disease. J. Clin. Microbiol. 41 (7), 2915–2923. doi: 10.1128/JCM.41.7.2915-2923.2003 12843021 PMC165291

[B7] Cao Y. Oh J. Xue M. Huh W. J. Wang J. Gonzalez-Hernandez J. A.. (2022). Commensal microbiota from patients with inflammatory bowel disease produce genotoxic metabolites. Science 378 (6618), eabm3233. doi: 10.1126/science.abm3233 36302024 PMC9993714

[B8] Chehoud C. Albenberg L. G. Judge C. Hoffmann C. Grunberg S. Bittinger K.. (2015). Fungal signature in the gut microbiota of pediatric patients with inflammatory bowel disease. Inflammation Bowel Dis. 21 (8), 1948–1956. doi: 10.1097/MIB.0000000000000454 PMC4509842 26083617

[B9] Chen X. Zhang D. Sun H. Jiang F. Shen Y. Wei P.. (2021). Characterization of the gut microbiota in Chinese children with overweight and obesity using 16S rRNA gene sequencing. PeerJ 9, e11439. doi: 10.7717/peerj.11439 34164233 PMC8194416

[B10] Chiaro T. R. Soto R. Zac Stephens W. Kubinak J. L. Petersen C. Gogokhia L.. (2017). A member of the gut mycobiota modulates host purine metabolism exacerbating colitis in mice. Sci. Transl. Med. 9 (380). doi: 10.1126/scitranslmed.aaf9044 PMC5994919 28275154

[B11] Clavien P. A. Barkun J. de Oliveira M. L. Vauthey J. N. Dindo D. Schulick R. D.. (2009). The Clavien-Dindo classification of surgical complications: five-year experience. Ann. Surg. 250 (2), 187–196. doi: 10.1097/SLA.0b013e3181b13ca2 19638912

[B12] Dindo D. Demartines N. Clavien P.-A. (2004). Classification of surgical complications: a new proposal with evaluation in a cohort of 6336 patients and results of a survey. Ann. Surg. 240 (2), 205–213. doi: 10.1097/01.sla.0000133083.54934.ae 15273542 PMC1360123

[B13] Duan X. Wu T. Xu X. Chen D. Mo A. Lei Y.. (2017). Smoking may lead to marginal bone loss around non-submerged implants during bone healing by altering salivary microbiome: A prospective study. J. Periodontol 88 (12), 1297–1308. doi: 10.1902/jop.2017.160808 28844190

[B14] Frank D. N. St Amand A. L. Feldman R. A. Boedeker E. C. Harpaz N. Pace N. R. (2007). Molecular-phylogenetic characterization of microbial community imbalances in human inflammatory bowel diseases. Proc. Natl. Acad. Sci. U.S.A. 104 (34), 13780–13785. doi: 10.1073/pnas.0706625104 17699621 PMC1959459

[B15] Galazzo G. Tedjo D. I. Wintjens D. S. J. Savelkoul P. H. M. Masclee A. A. M. Bodelier A. G. L.. (2019). Faecal microbiota dynamics and their relation to disease course in Crohn's disease. J. Crohns Colitis 13 (10), 1273–1282. doi: 10.1093/ecco-jcc/jjz049 30810207 PMC6764104

[B16] Goodrich J. K. Waters J. L. Poole A. C. Sutter J. L. Koren O. Blekhman R.. (2014). Human genetics shape the gut microbiome. Cell 159 (4), 789–799. doi: 10.1016/j.cell.2014.09.053 25417156 PMC4255478

[B17] Guberna L. Nyssen O. P. Chaparro M. Gisbert J. P. (2021). Frequency and effectiveness of empirical anti-TNF dose intensification in inflammatory bowel disease: systematic review with meta-analysis. J. Clin. Med. 10 (10), 2132. doi: 10.3390/jcm10102132 34069295 PMC8156358

[B18] Guyton K. Alverdy J. C. (2017). The gut microbiota and gastrointestinal surgery. Nat. Rev. Gastroenterol. Hepatol. 14 (1), 43–54. doi: 10.1038/nrgastro.2016.139 27729657

[B19] Ha C. W. Y. Martin A. Sepich-Poore G. D. Shi B. Wang Y. Gouin K.. (2020). Translocation of viable gut microbiota to mesenteric adipose drives formation of creeping fat in humans. Cell 183 (3), 666–683.e17. doi: 10.1016/j.cell.2020.09.009 32991841 PMC7521382

[B20] Ihrmark K. Bödeker I. T. M. Cruz-Martinez K. Friberg H. Kubartova A. Schenck J.. (2012). New primers to amplify the fungal ITS2 region–evaluation by 454-sequencing of artificial and natural communities. FEMS Microbiol. Ecol. 82 (3), 666–677. doi: 10.1111/j.1574-6941.2012.01437.x 22738186

[B21] Kelesidis T. Pothoulakis C. (2012). Efficacy and safety of the probiotic Saccharomyces boulardii for the prevention and therapy of gastrointestinal disorders. Therap Adv. Gastroenterol. 5 (2), 111–125. doi: 10.1177/1756283X11428502 PMC3296087 22423260

[B22] Klindworth A. Pruesse E. Schweer T. Peplies J. Quast C. Horn M.. (2013). Evaluation of general 16S ribosomal RNA gene PCR primers for classical and next-generation sequencing-based diversity studies. Nucleic Acids Res. 41 (1), e1. doi: 10.1093/nar/gks808 22933715 PMC3592464

[B23] Kõljalg U. Nilsson H. R. Schigel D. Tedersoo L. Larsson K.-H. May T. W.. (2020). The taxon hypothesis paradigm-on the unambiguous detection and communication of taxa. Microorganisms 8 (12), 1910. doi: 10.3390/microorganisms8121910 33266327 PMC7760934

[B24] Kotlowski R. Bernstein C. N. Sepehri S. Krause D. O. (2007). High prevalence of Escherichia coli belonging to the B2+D phylogenetic group in inflammatory bowel disease. Gut 56 (5), 669–675. doi: 10.1136/gut.2006.099796 17028128 PMC1942160

[B25] Kozich J. J. Westcott S. L. Baxter N. T. Highlander S. K. Schloss P. D. (2013). Development of a dual-index sequencing strategy and curation pipeline for analyzing amplicon sequence data on the MiSeq Illumina sequencing platform. Appl. Environ. Microbiol. 79 (17), 5112–5120. doi: 10.1128/AEM.01043-13 23793624 PMC3753973

[B26] Lederer A.-K. Chikhladze S. Kohnert E. Huber R. Müller A. (2021). Current insights: the impact of gut microbiota on postoperative complications in visceral surgery-A narrative review. Diagnostics (Basel) 11 (11), 2099. doi: 10.3390/diagnostics11112099 34829446 PMC8625751

[B27] Lee M. Chang E. B. (2021). Inflammatory bowel diseases (IBD) and the microbiome-searching the crime scene for clues. Gastroenterology 160 (2), 524–537. doi: 10.1053/j.gastro.2020.09.056 33253681 PMC8098834

[B28] Li E. Zhang Y. Tian X. Wang X. Gathungu G. Wolber A.. (2019). Influence of Crohn's disease related polymorphisms in innate immune function on ileal microbiome. PLoS One 14 (2), e0213108. doi: 10.1371/journal.pone.0213108 30818349 PMC6395037

[B29] Liguori G. Lamas B. Richard M. L. Brandi G. Da Costa G. Hoffmann T. W.. (2016). Fungal dysbiosis in mucosa-associated microbiota of Crohn's disease patients. J. Crohns Colitis 10 (3), 296–305. doi: 10.1093/ecco-jcc/jjv209 26574491 PMC4957473

[B30] Limon J. J. Tang J. Li D. Wolf A. J. Michelsen K. S. Funari V.. (2019). Malassezia is associated with Crohn's disease and exacerbates colitis in mouse models. Cell Host Microbe 25 (3), 377–388.e6. doi: 10.1016/j.chom.2019.01.007 30850233 PMC6417942

[B31] Macfarlane G. T. Macfarlane S. (2011). Fermentation in the human large intestine: its physiologic consequences and the potential contribution of prebiotics. J. Clin. Gastroenterol. 45 Suppl, S120–S127. doi: 10.1097/MCG.0b013e31822fecfe 21992950

[B32] Manichanh C. Borruel N. Casellas F. Guarner F. (2012). The gut microbiota in IBD. Nat. Rev. Gastroenterol. Hepatol. 9 (10), 599–608. doi: 10.1038/nrgastro.2012.152 22907164

[B33] Manichanh C. Rigottier-Gois L. Bonnaud E. Gloux K. Pelletier E. Frangeul L.. (2006). Reduced diversity of faecal microbiota in Crohn's disease revealed by a metagenomic approach. Gut 55 (2), 205–211. doi: 10.1136/gut.2005.073817 16188921 PMC1856500

[B34] Meng L. Xu M. Xing Y. Chen C. Jiang J. Xu X. (2022). Effects of cigarette smoke exposure on the gut microbiota and liver transcriptome in mice reveal gut-liver interactions. Int. J. Mol. Sci. 23 (19), 11008. doi: 10.3390/ijms231911008 36232309 PMC9569613

[B35] Naser S. A. Ghobrial G. Romero C. Valentine J. F. (2004). Culture of Mycobacterium avium subspecies paratuberculosis from the blood of patients with Crohn's disease. Lancet 364 (9439), 1039–1044. doi: 10.1016/S0140-6736(04)17058-X 15380962

[B36] Pryde S. E. Duncan S. H. Hold G. L. Stewart C. S. Flint H. J. (2002). The microbiology of butyrate formation in the human colon. FEMS Microbiol. Lett. 217 (2), 133–139. doi: 10.1111/j.1574-6968.2002.tb11467.x 12480096

[B37] Qin J. Li R. Raes J. Arumugam M. Burgdorf K. S. Manichanh C.. (2010). A human gut microbial gene catalogue established by metagenomic sequencing. Nature 464 (7285), 59–65. doi: 10.1038/nature08821 20203603 PMC3779803

[B38] Ricanek P. Lothe S. M. Frye S. A. Rydning A. Vatn M. H. Tønjum T. (2012). Gut bacterial profile in patients newly diagnosed with treatment-naïve Crohn's disease. Clin. Exp. Gastroenterol. 5, 173–186. doi: 10.2147/CEG.S33858 23049264 PMC3459595

[B39] Rinke C. ChuvoChina M. Mussig A. J. Chaumeil P.-A. Davín A. A. Waite D. W.. (2021). A standardized archaeal taxonomy for the Genome Taxonomy Database. Nat. Microbiol. 6 (7), 946–959. doi: 10.1038/s41564-021-00918-8 34155373

[B40] Ryan F. J. Ahern A. M. Fitzgerald R. S. Laserna-Mendieta E. J. Power E. M. Clooney A. G.. (2020). Colonic microbiota is associated with inflammation and host epigenomic alterations in inflammatory bowel disease. Nat. Commun. 11 (1), 1512. doi: 10.1038/s41467-020-15342-5 32251296 PMC7089947

[B41] Šašala M. Majorová E. Vrzgula A. Fandáková I. (2020). Evaluation of invasive intra-abdominal candidiasis in Crohn disease at the time of surgery. Ann. Coloproctol 36 (1), 12–16. doi: 10.3393/ac.2018.10.15.2 32146783 PMC7069678

[B42] Schmitt F. C. F. Brenner T. Uhle F. Loesch S. Hackert T. Ulrich A.. (2019). Gut microbiome patterns correlate with higher postoperative complication rates after pancreatic surgery. BMC Microbiol. 19 (1), 42. doi: 10.1186/s12866-019-1399-5 30777006 PMC6379976

[B43] Schwenk W. (2022). Optimized perioperative management (fast-track, ERAS) to enhance postoperative recovery in elective colorectal surgery. GMS Hyg Infect. Control 17, Doc10. doi: 10.3205/dgkh000413 35909653 PMC9284431

[B44] Sha S. Xu B. Wang X. Zhang Y. Wang H. Kong X.. (2013). The biodiversity and composition of the dominant fecal microbiota in patients with inflammatory bowel disease. Diagn. Microbiol. Infect. Dis. 75 (3), 245–251. doi: 10.1016/j.diagmicrobio.2012.11.022 23276768

[B45] Sokol H. Leducq V. Aschard H. Pham H.-P. Jegou S. Landman C.. (2017). Fungal microbiota dysbiosis in IBD. Gut 66 (6), 1039–1048. doi: 10.1136/gutjnl-2015-310746 26843508 PMC5532459

[B46] Sokol H. Pigneur B. Watterlot L. Lakhdari O. Bermúdez-Humarán L. G. Gratadoux J.-J.. (2008). Faecalibacterium prausnitzii is an anti-inflammatory commensal bacterium identified by gut microbiota analysis of Crohn disease patients. Proc. Natl. Acad. Sci. U.S.A. 105 (43), 16731–16736. doi: 10.1073/pnas.0804812105 18936492 PMC2575488

[B47] Standaert-Vitse A. Sendid B. Joossens M. François N. Vandewalle-El Khoury P. Branche J.. (2009). Candida albicans colonization and ASCA in familial Crohn's disease. Am. J. Gastroenterol. 104 (7), 1745–1753. doi: 10.1038/ajg.2009.225 19471251

[B48] Tedersoo L. Anslan S. Bahram M. Põlme S. Riit T. Liiv I.. (2015). Shotgun metagenomes and multiple primer pair-barcode combinations of amplicons reveal biases in metabarcoding analyses of fungi. MykoKyes 10, 1–43. doi: 10.3897/mycokeys.10.4852

[B49] The German Society for Gastroenterology Digestive and Metabolic Diseases (DGVS) (2021). S3-Guideline Diagnosis and Therapy of Crohn's Disease (S3-Leitlinie Diagnostik und Therapie des Morbus Crohn) (Berlin, Germany: German Society for Gastroenterology and Digestive and Metabolic Diseases (DGVS)).

[B50] Torres J. Bonovas S. Doherty G. Kucharzik T. Gisbert J. P. Raine T.. (2020). ECCO guidelines on therapeutics in Crohn's disease: medical treatment. J. Crohns Colitis 14 (1), 4–22. doi: 10.1093/ecco-jcc/jjz180 31711158

[B51] Torres J. Mehandru S. Colombel J.-F. Peyrin-Biroulet L. (2017). Crohn's disease. Lancet 389 (10080), 1741–1755. doi: 10.1016/S0140-6736(16)31711-1 27914655

[B52] Underhill D. M. Braun J. (2022). Fungal microbiome in inflammatory bowel disease: a critical assessment. J. Clin. Invest. 132 (5), e155786. doi: 10.1172/JCI155786 35229726 PMC8884899

[B53] Vasudevan A. Gibson P. R. van Langenberg D. R. (2017). Time to clinical response and remission for therapeutics in inflammatory bowel diseases: What should the clinician expect, what should patients be told? World J. Gastroenterol. 23 (35), 6385–6402. doi: 10.3748/wjg.v23.i35.6385 29085188 PMC5643264

[B54] Vermeire S. Peeters M. Vlietinck R. Joossens S. den Hond E. Bulteel V.. (2001). Anti-Saccharomyces cerevisiae antibodies (ASCA), phenotypes of IBD, and intestinal permeability: a study in IBD families. Inflammation Bowel Dis. 7 (1), 8–15. doi: 10.1097/00054725-200102000-00002 11233666

[B55] Yan S. Ma Z. Jiao M. Wang Y. Li A. Ding S. (2021). Effects of smoking on inflammatory markers in a healthy population as analyzed *via* the gut microbiota. Front. Cell Infect. Microbiol. 11, 633242. doi: 10.3389/fcimb.2021.633242 34368009 PMC8342938

[B56] Zheng L. Wen X.-L. Duan S.-L. (2022). Role of metabolites derived from gut microbiota in inflammatory bowel disease. World J. Clin. cases 10 (9), 2660–2677. doi: 10.12998/wjcc.v10.i9.2660 35434116 PMC8968818

