# Supplementary material for: Longitudinal dynamics of gut bacteriome and mycobiome interactions pre- and post-visceral surgery in Crohn’s disease
Source: Front Cell Infect Microbiol. 2024 Jan 15;13:1275405. doi: 10.3389/fcimb.2023.1275405 (PMC10822897; doi:10.3389/fcimb.2023.1275405)
Supplement: Supplementary file 1 [file Presentation_1.zip › Supplements_Crohn_Paper_Review_2.docx]

Supplementary Material

Longitudinal Dynamics of Gut Bacteriome and Mycobiome Interactions Pre- and Post-Visceral Surgery in Crohn's Disease

Simon Wetzel*****, Alexander Müller, Eva Kohnert, Negin Mehrbarzin, Roman Huber, Georg Häcker, Clemens Kreutz, Ann-Kathrin Lederer, Mohamed Tarek Badr

*** Correspondence:** Simon Wetzel: [simon.wetzel@web.de](mailto:simon.wetzel@web.de)


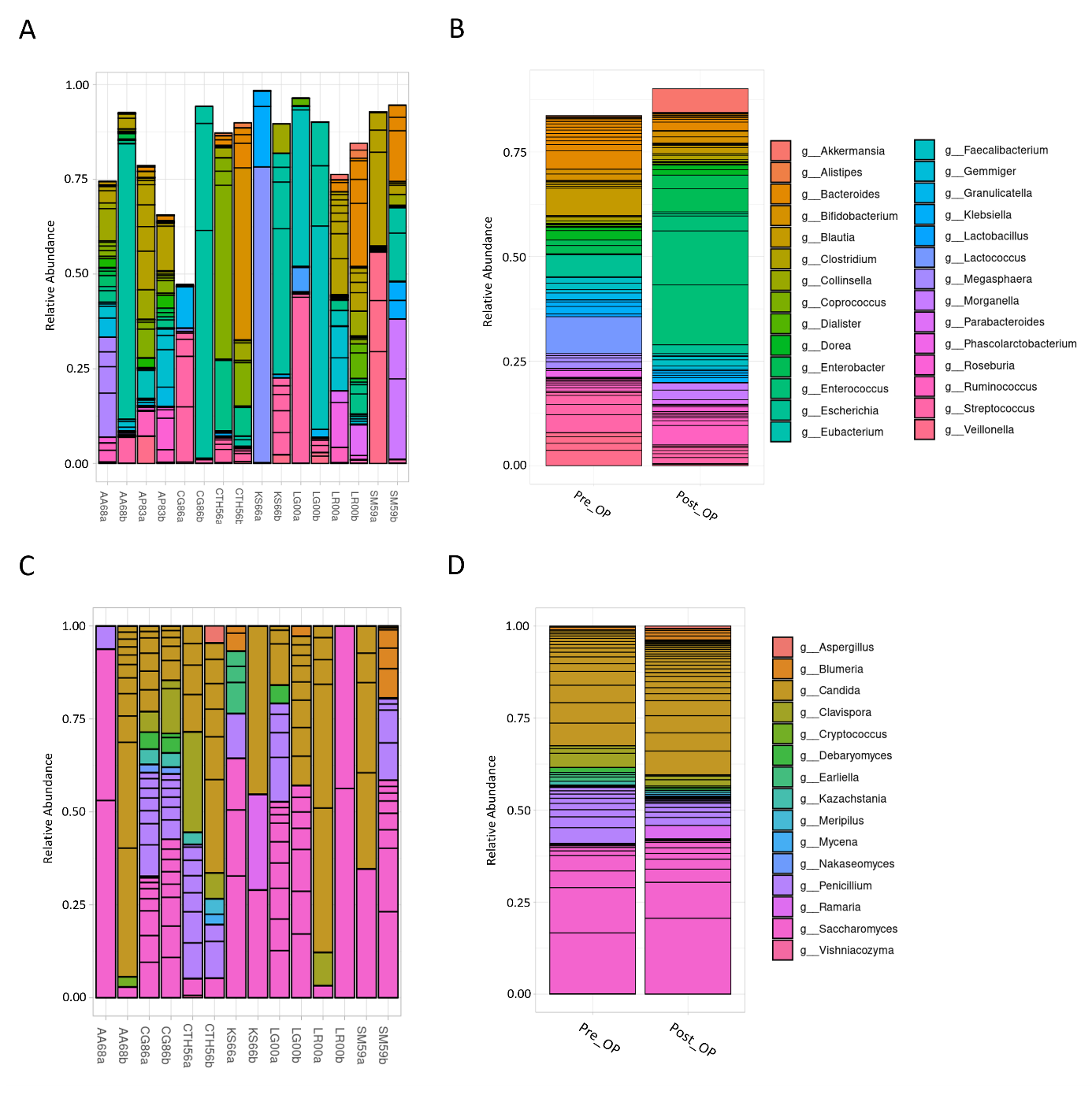


Supplementary Figure 1: Pre- to postoperative changes of bacterial and fungal genera
(A) Bacterial relative abundances on genus level ordered by each sample. Sample names with the suffix "a" represents preoperative samples, whereas “b” represents postoperative samples. (D) Comparison of bacterial relative abundances on genus level of merged preoperative and postoperative fecal samples. (C) Fungal relative abundances ordered by each sample. Sample names with the suffix "a" represents preoperative samples, whereas “b” represents postoperative samples. (D) Comparison of fungal relative abundances of merged preoperative and postoperative fecal samples.
Plots were generated after removing unassigned reads on genus level.


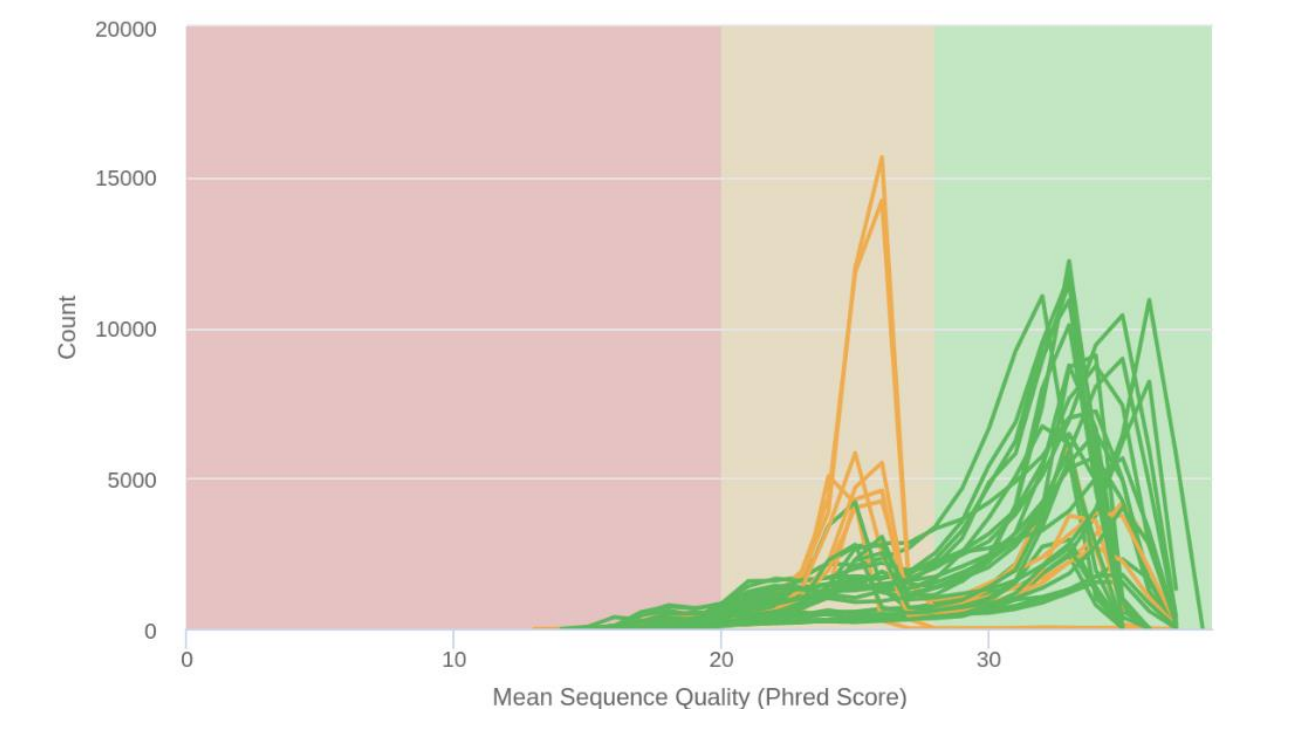


Supplementary Figure 2: Fungal ITS2 sequence quality plot
Plot was created using FastQC and MultiQC, green area indicates good quality score. Each line represents one sample.


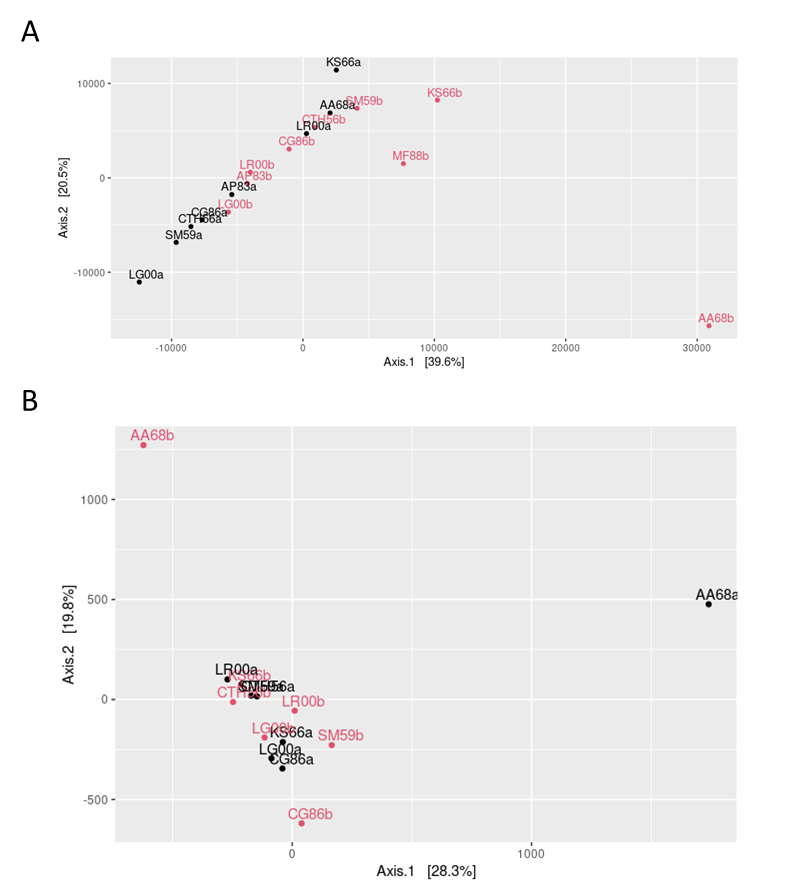


Supplementary Figure 3: Bacterial (A) and fungal (B) principal coordinate analysis (PCoA) measuring Euclidean distances of pre-and postoperative samples
Preoperative samples are highlighted in black and postoperative samples in red.


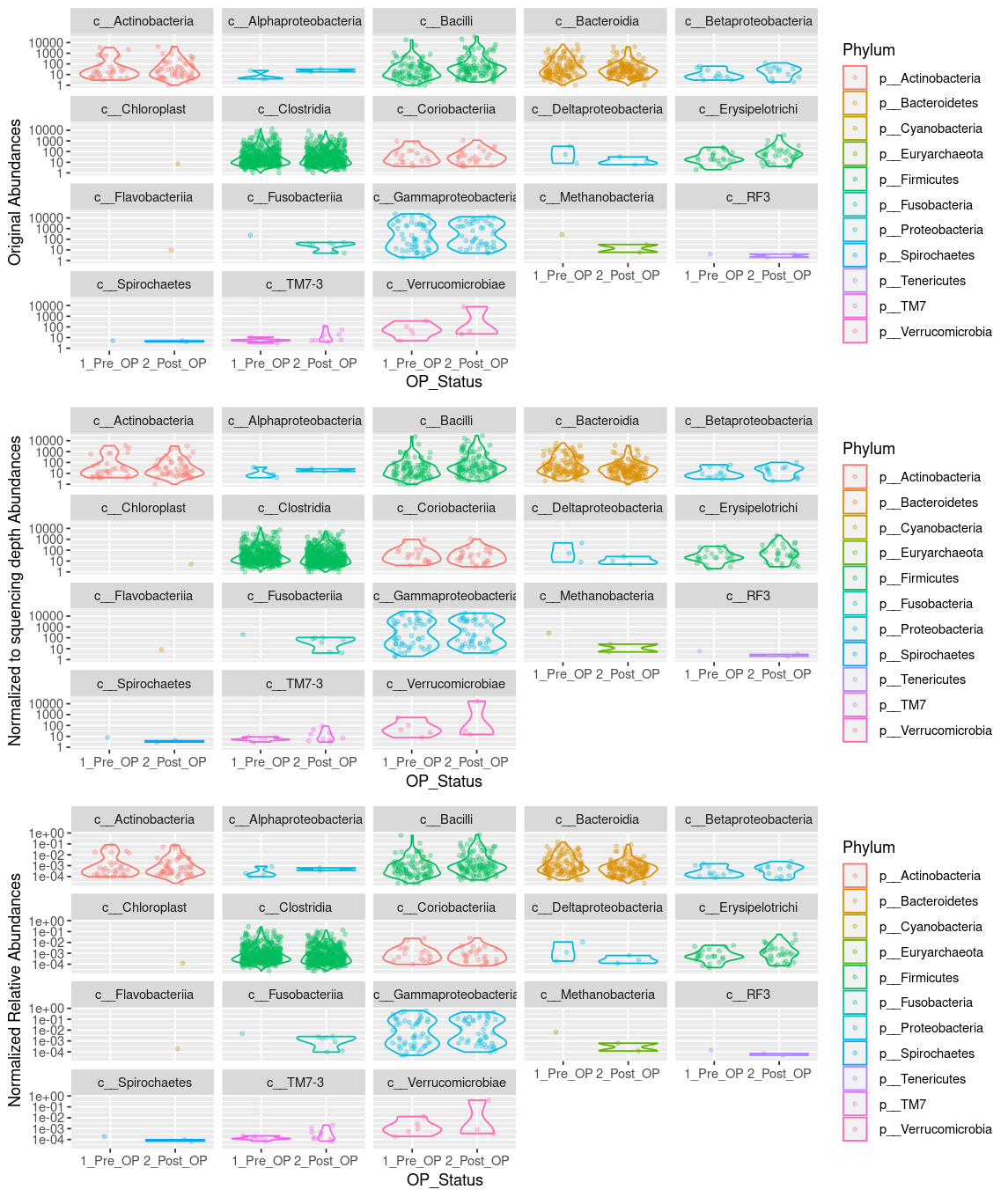


Supplementary Figure 4: Pre- and postsurgical bacterial abundances on class level
Visualized are original absolute abundances (top panel), normalized to sequencing depth abundances (mid panel) and normalized relative abundances (bottom panel). Plots were generated by using phyloseq.


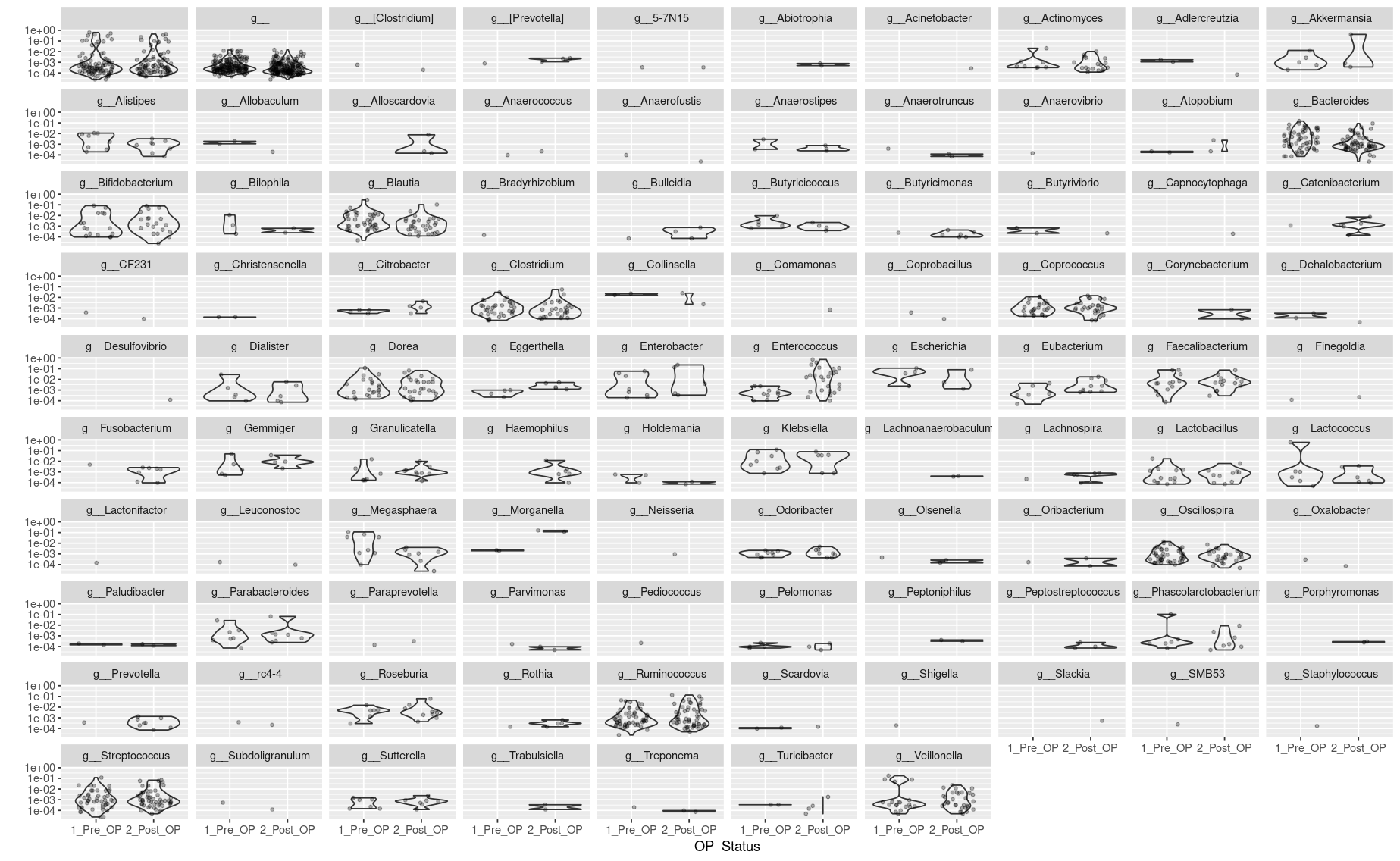


Supplementary Figure 5: Pre- and postsurgical bacterial relative abundances on genus level
Illustrated are all identified genera after initial filtering of sequenced bacterial reads. Plot was generated using the DADA 1.8 pipeline in the R programming language.


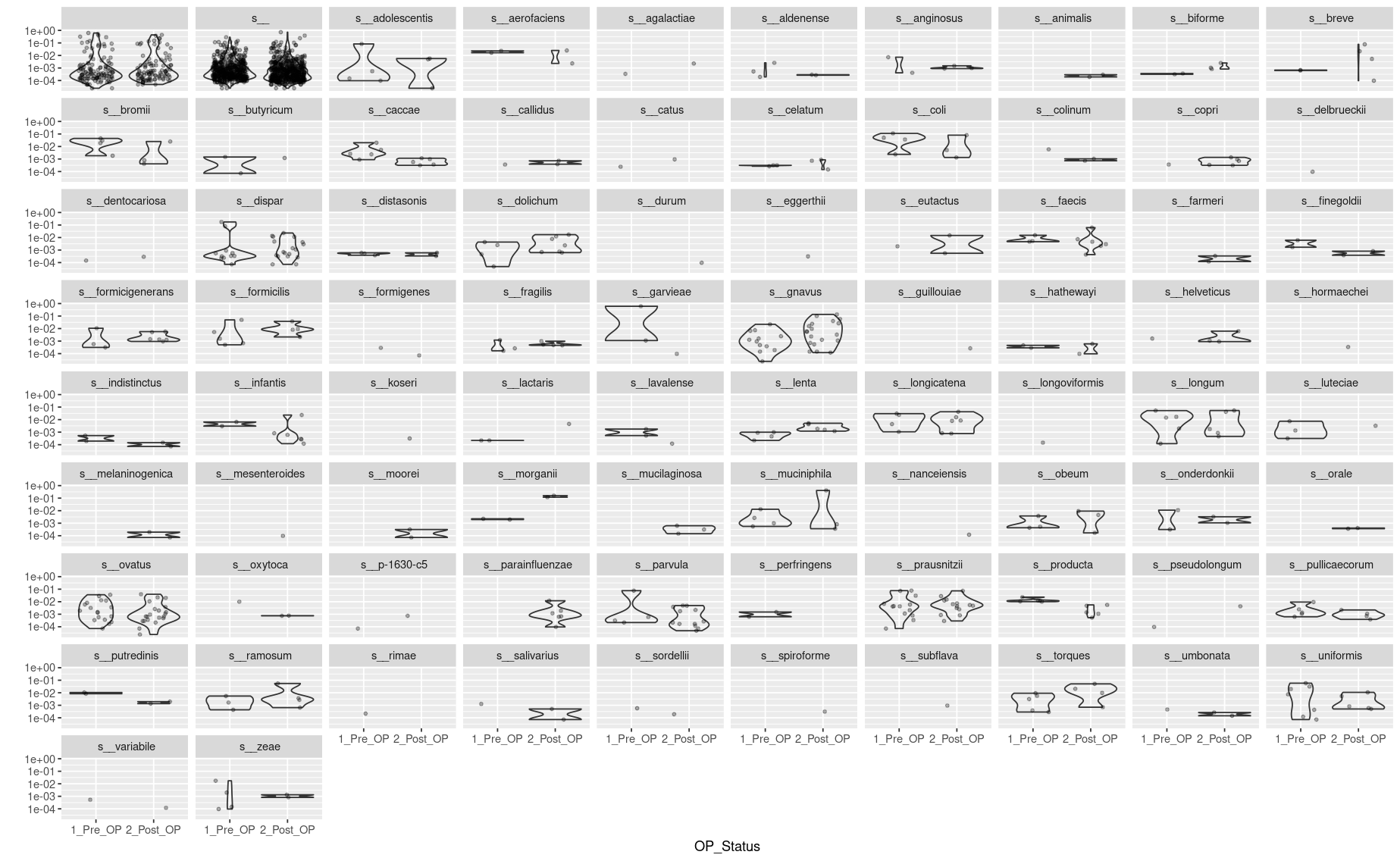


Supplementary Figure 6: Pre- and postsurgical bacterial relative abundances on species level
Illustrated are all identified species after initial filtering of sequenced bacterial reads. Plot was generated using the DADA 1.8 pipeline in the R programming language.


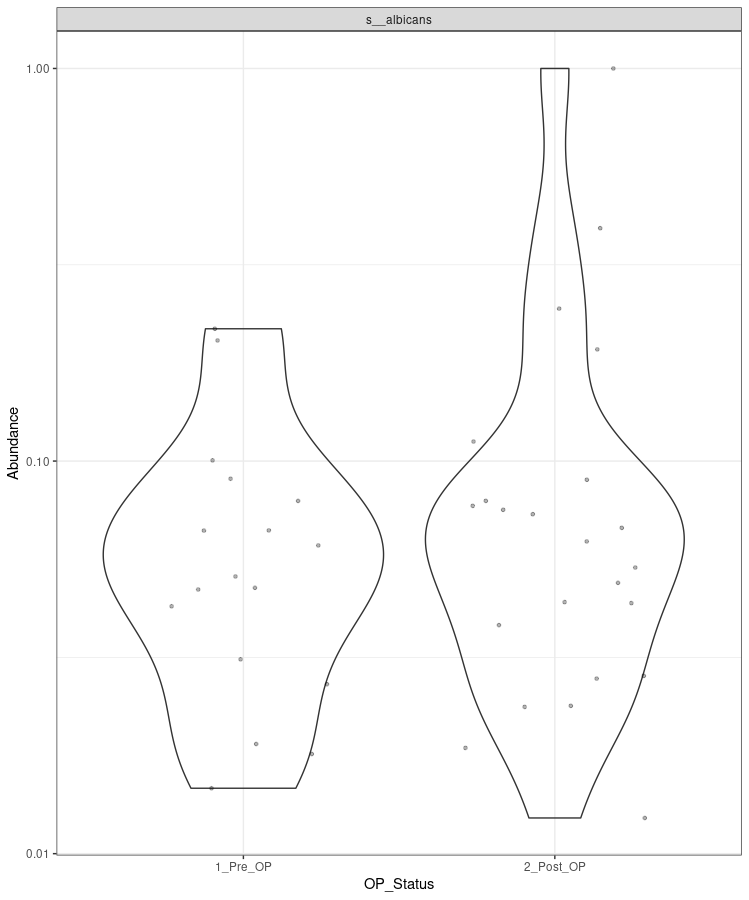


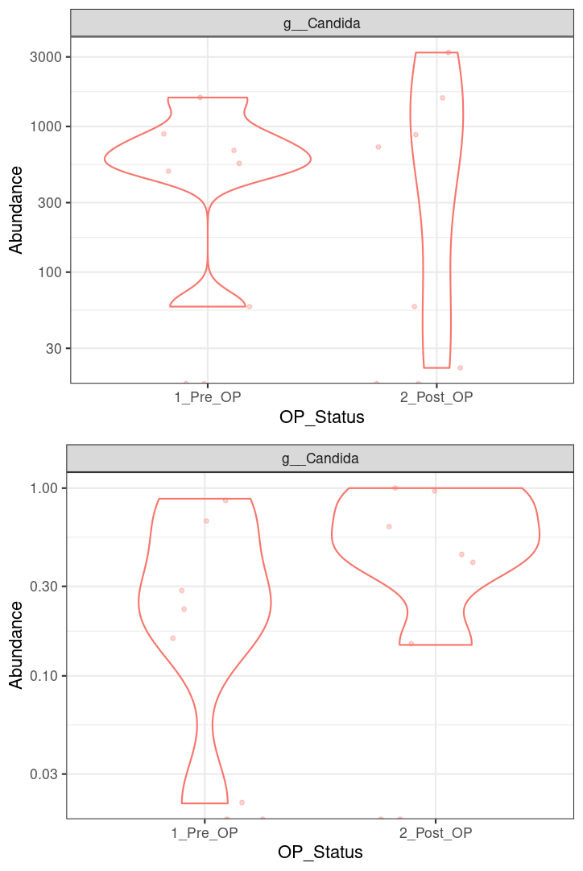


Supplementary Figure 7: Pre- and postsurgical relative abundances of Candida genus and *Candida albicans* species
Violin plot of relative abundances of the Candida genus and Candida albicans species in pre- and postsurgical samples. Plot was generated using the DADA 1.8 pipeline in the R programming language.


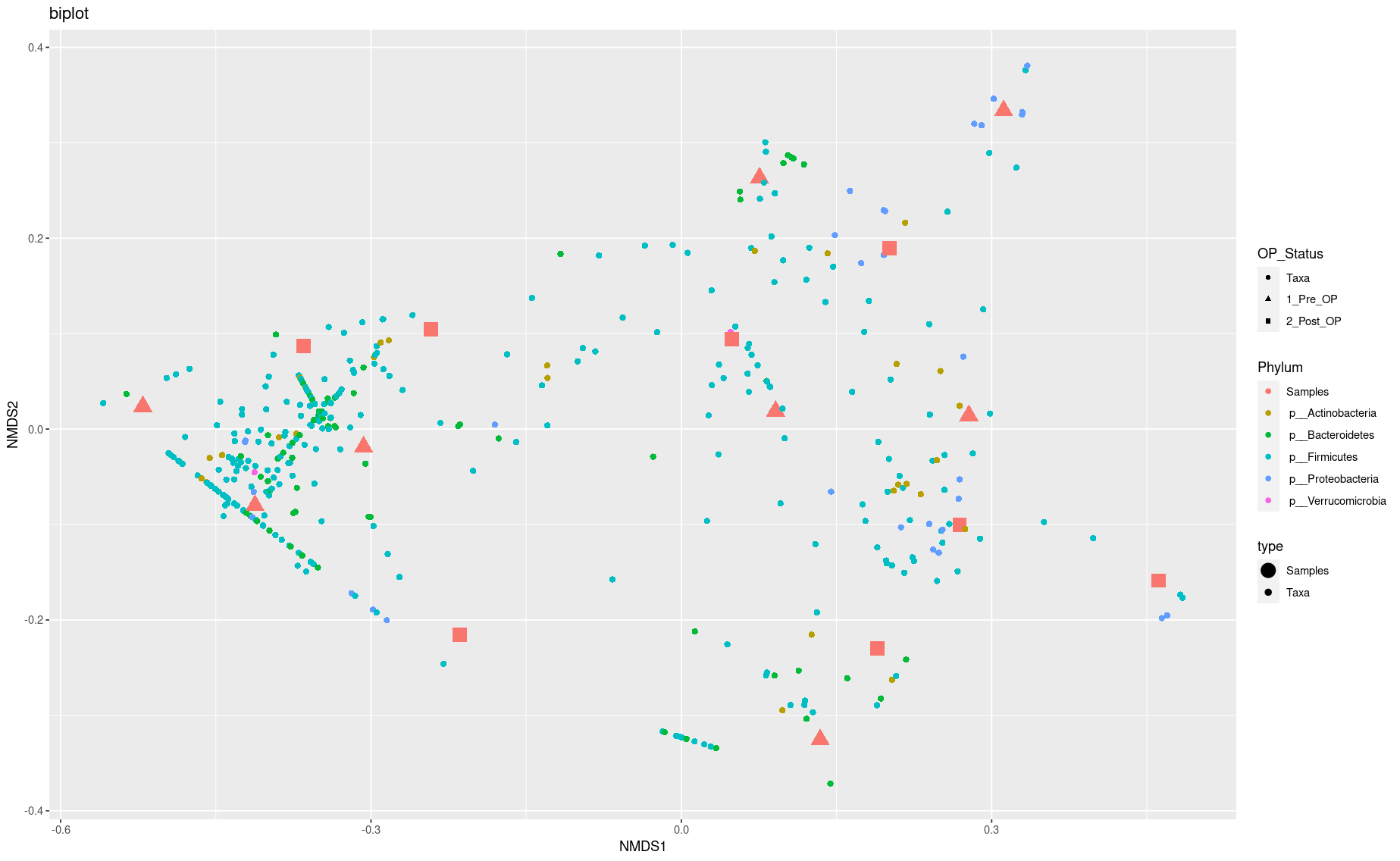


Supplementary Figure 8: Bacterial NMDS analysis with labelling of identified bacterial phyla and surgical status
Bray-Curtis distances in bacterial beta diversity between pre- and postsurgical samples were tested for significance by adonis testing.


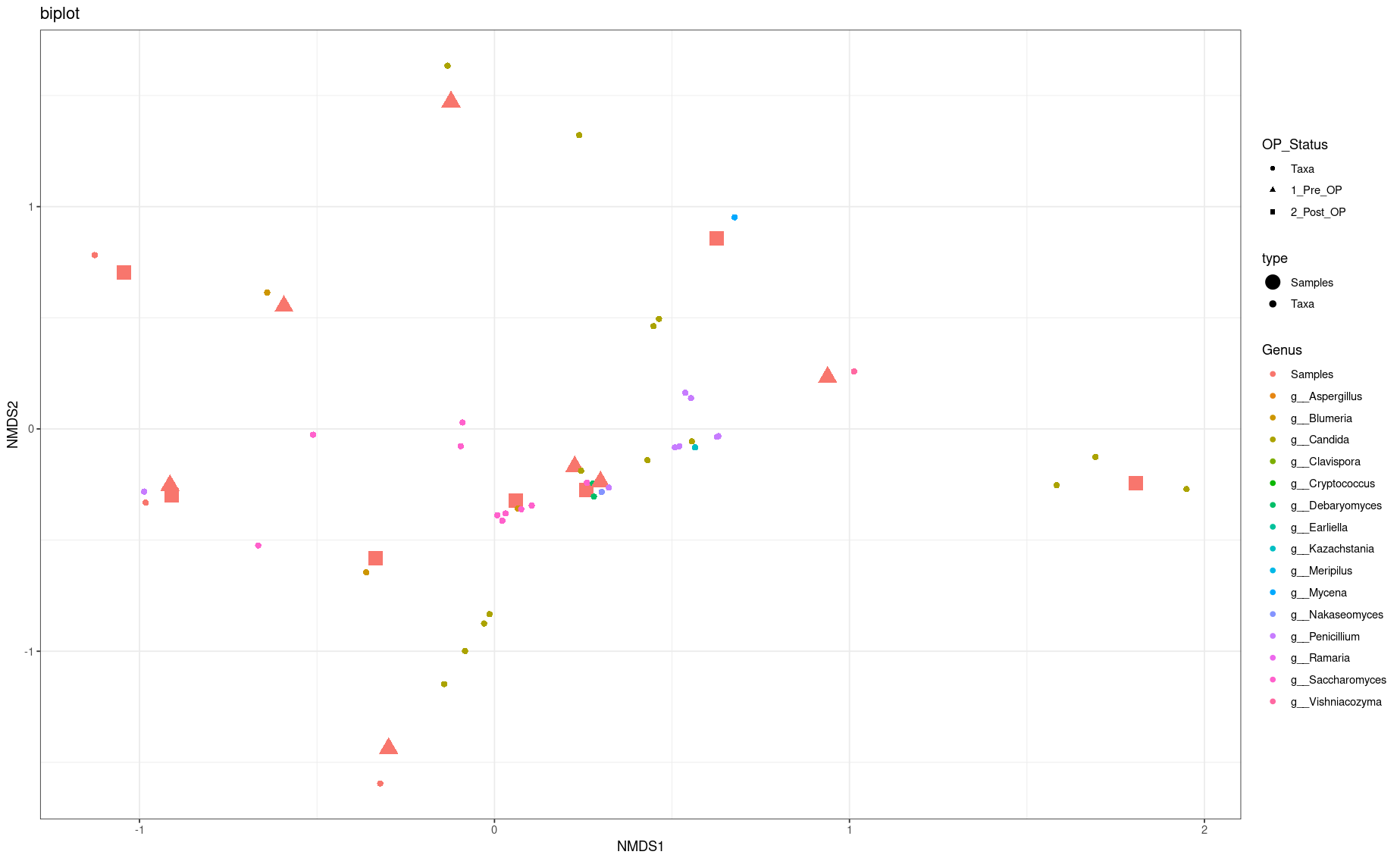


Supplementary Figure 9: Mycobial NMDS analysis with labelling of identified mycobial genera and surgical status
Bray-Curtis distances in mycobial beta diversity between pre- and postsurgical samples were tested for significance by adonis testing.


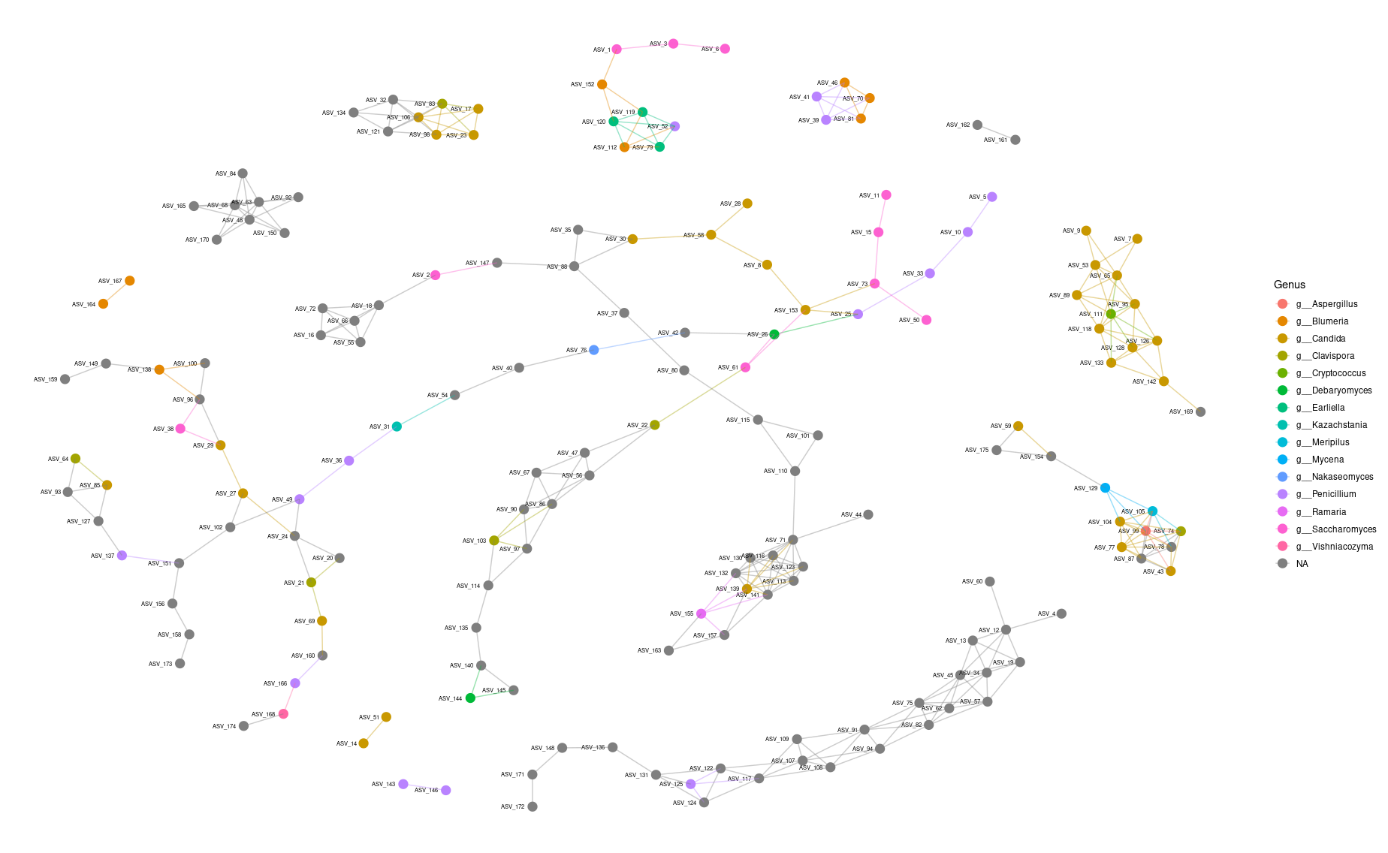


Supplementary Figure 10: Mycobial interaction network analysis
Labelling of mycobial ASVs on genus level. Plot was generated using SpiecEasi and phyloseq packages in R programming language.


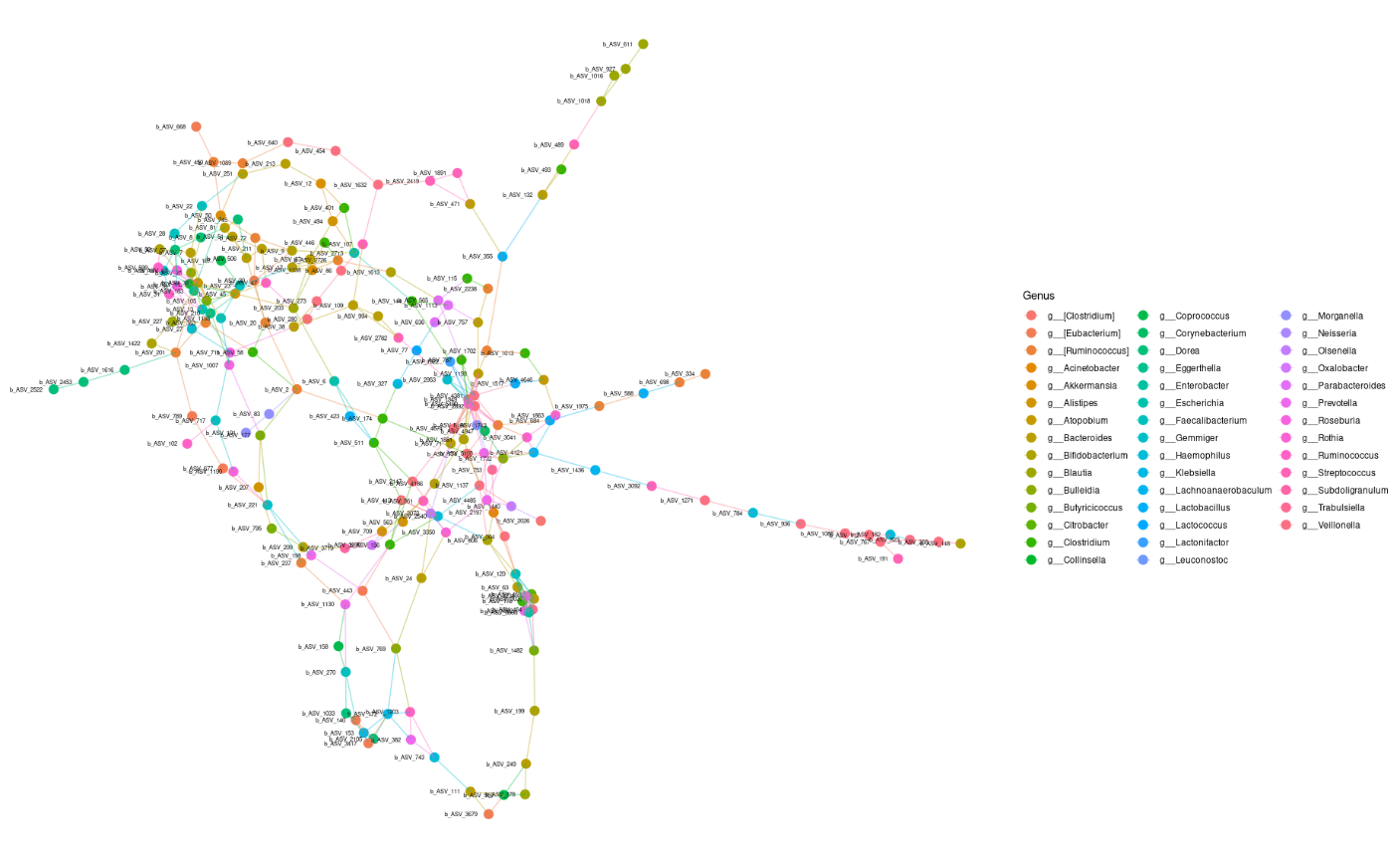


Supplementary Figure 11: Bacterial interaction network analysis
Labelling of bacterial ASVs on genus level. Plot was generated using SpiecEasi and phyloseq packages in R programming language.

*In three cases ASVs belonging to the same genus were assigned to two genera (g__[Clostridium], g__[Eubacterium],g__[Ruminococcus]) by taxonomic assignment of reference database*


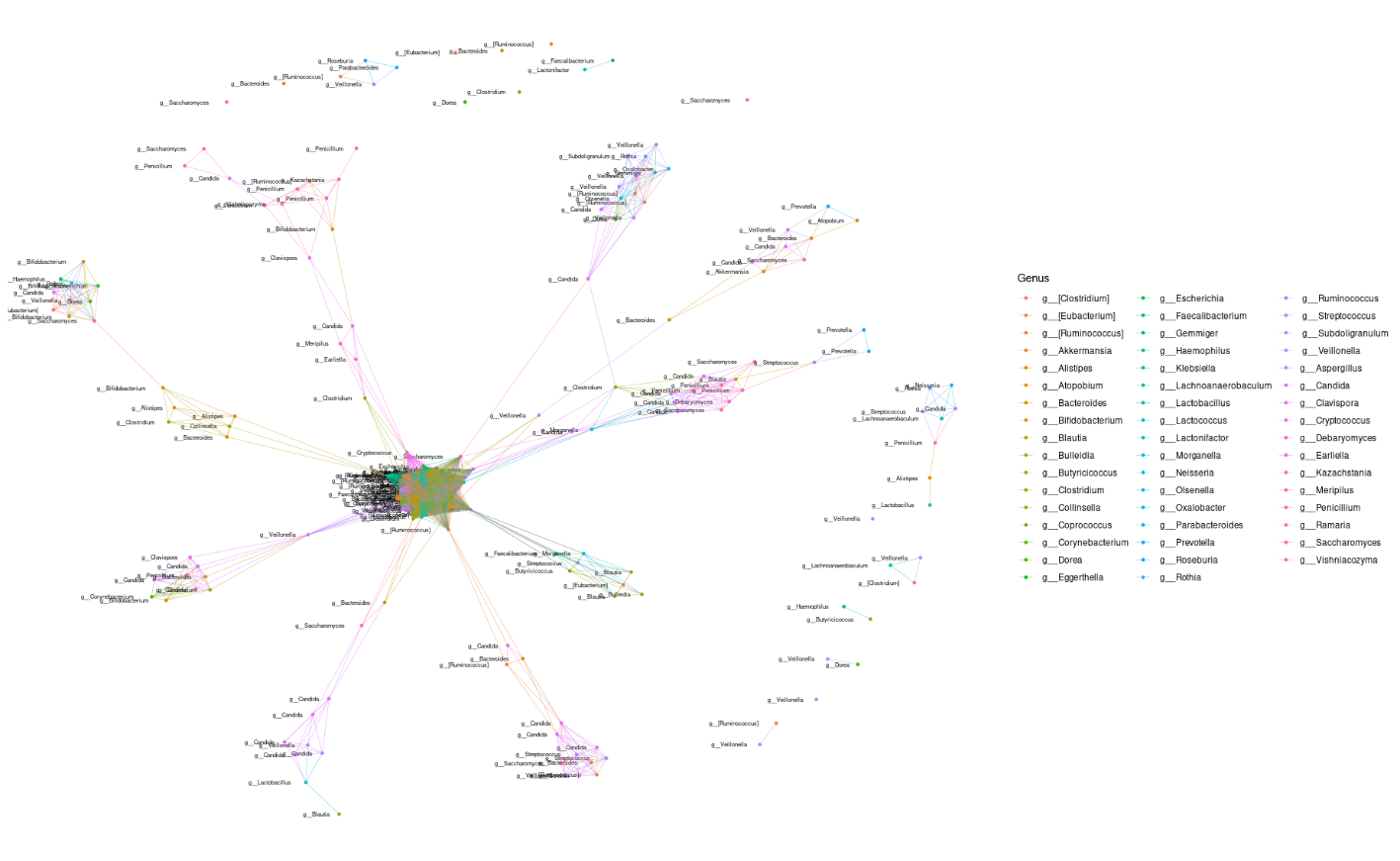


Supplementary Figure 12: Mycobiome-bacteriome interaction network analysis
Labelling of bacterial and mycobial ASVs on genus level. Plot was generated using SpiecEasi and phyloseq packages in R programming language.

*In three cases ASVs belonging to the same genus were assigned to two genera (g__[Clostridium], g__[Eubacterium],g__[Ruminococcus]) by taxonomic assignment of reference database*

**Supplementary Table 1: Louvian clustering output**

| **Nodes_1** | **Cluster _ID_1** | **Nodes_2** | **Cluster _ID_2** | **Nodes_3** | **Cluster _ID_3** | **Nodes_4** | **Cluster _ID_4** | **Nodes_5** | **Cluster _ID_5** |
| --- | --- | --- | --- | --- | --- | --- | --- | --- | --- |
| ASV_3 | 3 | ASV_118 | 6 | b_ASV_511 | 6 | b_ASV_636 | 6 | b_ASV_4485 | 11 |
| ASV_7 | 3 | ASV_119 | 6 | b_ASV_563 | 6 | ASV_17 | 7 | b_ASV_4646 | 11 |
| ASV_9 | 3 | ASV_143 | 6 | b_ASV_588 | 6 | ASV_98 | 7 | ASV_74 | 12 |
| ASV_10 | 3 | ASV_155 | 6 | b_ASV_599 | 6 | b_ASV_745 | 7 | ASV_85 | 12 |
| ASV_26 | 3 | ASV_166 | 6 | b_ASV_606 | 6 | b_ASV_767 | 7 | ASV_104 | 12 |
| ASV_28 | 3 | b_ASV_6 | 6 | b_ASV_611 | 6 | b_ASV_1440 | 7 | ASV_106 | 12 |
| ASV_29 | 3 | b_ASV_8 | 6 | b_ASV_636 | 6 | b_ASV_1702 | 7 | ASV_137 | 12 |
| ASV_33 | 3 | b_ASV_12 | 6 | ASV_17 | 7 | b_ASV_2147 | 7 | b_ASV_58 | 12 |
| ASV_38 | 3 | b_ASV_14 | 6 | ASV_98 | 7 | b_ASV_2238 | 7 | b_ASV_81 | 12 |
| ASV_41 | 3 | b_ASV_22 | 6 | b_ASV_745 | 7 | b_ASV_2892 | 7 | b_ASV_112 | 12 |
| ASV_65 | 3 | b_ASV_23 | 6 | b_ASV_767 | 7 | b_ASV_2953 | 7 | b_ASV_4186 | 12 |
| b_ASV_25 | 3 | b_ASV_28 | 6 | b_ASV_1440 | 7 | b_ASV_3350 | 7 | b_ASV_4947 | 12 |
| b_ASV_101 | 3 | b_ASV_31 | 6 | b_ASV_1702 | 7 | b_ASV_3719 | 7 | b_ASV_13 | 14 |
| b_ASV_144 | 3 | b_ASV_45 | 6 | b_ASV_2147 | 7 | b_ASV_3990 | 7 | b_ASV_30 | 14 |
| b_ASV_3092 | 3 | b_ASV_51 | 6 | b_ASV_2238 | 7 | b_ASV_4381 | 7 | b_ASV_83 | 14 |
| b_ASV_5160 | 3 | b_ASV_57 | 6 | b_ASV_2892 | 7 | ASV_27 | 8 | b_ASV_105 | 14 |
| b_ASV_5490 | 3 | b_ASV_68 | 6 | b_ASV_2953 | 7 | ASV_58 | 8 | b_ASV_107 | 14 |
| ASV_5 | 4 | b_ASV_71 | 6 | b_ASV_3350 | 7 | ASV_69 | 8 | b_ASV_927 | 14 |
| ASV_6 | 4 | b_ASV_72 | 6 | b_ASV_3719 | 7 | ASV_73 | 8 | b_ASV_1016 | 14 |
| ASV_14 | 4 | b_ASV_77 | 6 | b_ASV_3990 | 7 | ASV_139 | 8 | b_ASV_1732 | 14 |
| ASV_22 | 4 | b_ASV_86 | 6 | b_ASV_4381 | 7 | ASV_142 | 8 | b_ASV_698 | 17 |
| ASV_25 | 4 | b_ASV_102 | 6 | ASV_27 | 8 | b_ASV_87 | 8 | b_ASV_774 | 17 |
| ASV_31 | 4 | b_ASV_109 | 6 | ASV_58 | 8 | b_ASV_1018 | 8 | b_ASV_1007 | 17 |
| ASV_39 | 4 | b_ASV_132 | 6 | ASV_69 | 8 | b_ASV_1088 | 8 | b_ASV_1113 | 17 |
| ASV_52 | 4 | b_ASV_148 | 6 | ASV_73 | 8 | b_ASV_1517 | 8 | b_ASV_1632 | 26 |
| ASV_105 | 4 | b_ASV_156 | 6 | ASV_139 | 8 | ASV_30 | 9 | b_ASV_2026 | 26 |
| ASV_111 | 4 | b_ASV_158 | 6 | ASV_142 | 8 | ASV_50 | 9 | b_ASV_4121 | 26 |
| ASV_120 | 4 | b_ASV_167 | 6 | b_ASV_87 | 8 | b_ASV_9 | 9 | b_ASV_4485 | 11 |
| ASV_125 | 4 | b_ASV_177 | 6 | b_ASV_1018 | 8 | b_ASV_11 | 9 | b_ASV_4646 | 11 |
| ASV_128 | 4 | b_ASV_182 | 6 | b_ASV_1088 | 8 | b_ASV_38 | 9 | ASV_74 | 12 |
| ASV_168 | 4 | b_ASV_183 | 6 | b_ASV_1517 | 8 | b_ASV_39 | 9 | ASV_85 | 12 |
| b_ASV_20 | 4 | b_ASV_191 | 6 | ASV_30 | 9 | b_ASV_115 | 9 | ASV_104 | 12 |
| b_ASV_52 | 4 | b_ASV_201 | 6 | ASV_50 | 9 | b_ASV_207 | 9 | ASV_106 | 12 |
| b_ASV_174 | 4 | b_ASV_209 | 6 | b_ASV_9 | 9 | b_ASV_789 | 9 | ASV_137 | 12 |
| ASV_8 | 5 | b_ASV_210 | 6 | b_ASV_11 | 9 | b_ASV_1271 | 9 | b_ASV_58 | 12 |
| ASV_15 | 5 | b_ASV_211 | 6 | b_ASV_38 | 9 | b_ASV_1422 | 9 | b_ASV_81 | 12 |
| ASV_23 | 5 | b_ASV_213 | 6 | b_ASV_39 | 9 | b_ASV_1613 | 9 | b_ASV_112 | 12 |
| ASV_59 | 5 | b_ASV_227 | 6 | b_ASV_115 | 9 | b_ASV_1863 | 9 | b_ASV_4186 | 12 |
| ASV_126 | 5 | b_ASV_251 | 6 | b_ASV_207 | 9 | b_ASV_1881 | 9 | b_ASV_4947 | 12 |
| ASV_133 | 5 | b_ASV_273 | 6 | b_ASV_789 | 9 | b_ASV_2453 | 9 | b_ASV_13 | 14 |
| b_ASV_2 | 5 | b_ASV_280 | 6 | b_ASV_1271 | 9 | b_ASV_2540 | 9 | b_ASV_30 | 14 |
| b_ASV_17 | 5 | b_ASV_304 | 6 | b_ASV_1422 | 9 | b_ASV_2713 | 9 | b_ASV_83 | 14 |
| b_ASV_823 | 5 | b_ASV_327 | 6 | b_ASV_1613 | 9 | ASV_51 | 10 | b_ASV_105 | 14 |
| b_ASV_994 | 5 | b_ASV_334 | 6 | b_ASV_1863 | 9 | ASV_146 | 10 | b_ASV_107 | 14 |
| b_ASV_1975 | 5 | b_ASV_355 | 6 | b_ASV_1881 | 9 | b_ASV_684 | 10 | b_ASV_927 | 14 |
| b_ASV_2419 | 5 | b_ASV_361 | 6 | b_ASV_2453 | 9 | b_ASV_709 | 10 | b_ASV_1016 | 14 |
| b_ASV_2782 | 5 | b_ASV_363 | 6 | b_ASV_2540 | 9 | b_ASV_1436 | 10 | b_ASV_1732 | 14 |
| ASV_11 | 6 | b_ASV_401 | 6 | b_ASV_2713 | 9 | b_ASV_1891 | 10 | b_ASV_698 | 17 |
| ASV_21 | 6 | b_ASV_412 | 6 | ASV_51 | 10 | b_ASV_3041 | 10 | b_ASV_774 | 17 |
| ASV_43 | 6 | b_ASV_423 | 6 | ASV_146 | 10 | b_ASV_3072 | 10 | b_ASV_1007 | 17 |
| ASV_64 | 6 | b_ASV_446 | 6 | b_ASV_684 | 10 | ASV_53 | 11 | b_ASV_1113 | 17 |
| ASV_77 | 6 | b_ASV_450 | 6 | b_ASV_709 | 10 | ASV_61 | 11 | b_ASV_1632 | 26 |
| ASV_79 | 6 | b_ASV_454 | 6 | b_ASV_1436 | 10 | ASV_153 | 11 | b_ASV_2026 | 26 |
| ASV_83 | 6 | b_ASV_471 | 6 | b_ASV_1891 | 10 | b_ASV_24 | 11 | b_ASV_4121 | 26 |
| ASV_89 | 6 | b_ASV_489 | 6 | b_ASV_3041 | 10 | b_ASV_50 | 11 | b_ASV_4485 | 11 |
| ASV_95 | 6 | b_ASV_493 | 6 | b_ASV_3072 | 10 | b_ASV_268 | 11 | b_ASV_4646 | 11 |
| ASV_99 | 6 | b_ASV_494 | 6 | ASV_53 | 11 | b_ASV_1137 | 11 | ASV_74 | 12 |
| ASV_103 | 6 | b_ASV_506 | 6 | ASV_61 | 11 | b_ASV_1198 | 11 | ASV_85 | 12 |
